# Extracellular Purine Metabolism Is the Switchboard of Immunosuppressive Macrophages and a Novel Target to Treat Diseases With Macrophage Imbalances

**DOI:** 10.3389/fimmu.2018.00852

**Published:** 2018-04-27

**Authors:** Anna Ohradanova-Repic, Christian Machacek, Celine Charvet, Franck Lager, Delphine Le Roux, René Platzer, Vladimir Leksa, Goran Mitulovic, Thomas R. Burkard, Gerhard J. Zlabinger, Michael B. Fischer, Vincent Feuillet, Gilles Renault, Stephan Blüml, Miroslav Benko, Miloslav Suchanek, Johannes B. Huppa, Takami Matsuyama, Artur Cavaco-Paulo, Georges Bismuth, Hannes Stockinger

**Affiliations:** ^1^Molecular Immunology Unit, Institute for Hygiene and Applied Immunology, Center for Pathophysiology, Infectiology and Immunology, Medical University of Vienna, Vienna, Austria; ^2^Institut National de la Santé et de la Recherche Médicale, INSERM U1016, Institut Cochin, Paris, France; ^3^Université Paris Descartes, Paris, France; ^4^Centre National de la Recherche Scientifique (CNRS), UMR 8104, Paris, France; ^5^Laboratory of Molecular Immunology, Institute of Molecular Biology, Slovak Academy of Sciences, Bratislava, Slovakia; ^6^Clinical Department of Medical and Chemical Laboratory Diagnostics, Medical University of Vienna, Vienna, Austria; ^7^Bioinformatics Department of the Research Institute of Molecular Pathology and the Institute of Molecular Biotechnology of the Austrian Academy of Sciences, Vienna, Austria; ^8^Institute of Immunology, Center for Pathophysiology, Infectiology and Immunology, Medical University of Vienna, Vienna, Austria; ^9^Department of Transfusion Medicine, Medical University of Vienna, Vienna, Austria; ^10^Center for Biomedical Technology, Danube University Krems, Krems, Austria; ^11^Division of Rheumatology, Internal Medicine III, Medical University of Vienna, Vienna, Austria; ^12^EXBIO Praha, Vestec, Czechia; ^13^The Center for Advanced Biomedical Sciences and Swine Research, Kagoshima University, Kagoshima, Japan; ^14^Centre of Biological Engineering, University of Minho, Campus of Gualtar, Braga, Portugal

**Keywords:** macrophage polarization, chronic inflammation, macrophage-T cell interaction, purine metabolism, adenosine, methotrexate, rheumatoid arthritis

## Abstract

If misregulated, macrophage (Mϕ)–T cell interactions can drive chronic inflammation thereby causing diseases, such as rheumatoid arthritis (RA). We report that in a proinflammatory environment, granulocyte-Mϕ (GM-CSF)- and Mϕ colony-stimulating factor (M-CSF)-dependent Mϕs have dichotomous effects on T cell activity. While GM-CSF-dependent Mϕs show a highly stimulatory activity typical for M1 Mϕs, M-CSF-dependent Mϕs, marked by folate receptor β (FRβ), adopt an immunosuppressive M2 phenotype. We find the latter to be caused by the purinergic pathway that directs release of extracellular ATP and its conversion to immunosuppressive adenosine by co-expressed CD39 and CD73. Since we observed a misbalance between immunosuppressive and immunostimulatory Mϕs in human and murine arthritic joints, we devised a new strategy for RA treatment based on targeted delivery of a novel methotrexate (MTX) formulation to the immunosuppressive FRβ^+^CD39^+^CD73^+^ Mϕs, which boosts adenosine production and curtails the dominance of proinflammatory Mϕs. In contrast to untargeted MTX, this approach leads to potent alleviation of inflammation in the murine arthritis model. In conclusion, we define the Mϕ extracellular purine metabolism as a novel checkpoint in Mϕ cell fate decision-making and an attractive target to control pathological Mϕs in immune-mediated diseases.

## Introduction

Macrophages (Mϕs) are myeloid immune cells essential for tissue homeostasis and immunity. Their differentiation and maintenance is controlled in a tissue-specific manner mainly by two growth factors, Mϕ and granulocyte-Mϕ colony-stimulating factor (M-CSF/CSF-1 and GM-CSF/CSF-2), respectively (1). In addition, the Mϕ phenotype is further shaped by local stimuli, to which Mϕs respond with high plasticity (2). During infection or sterile inflammation, proinflammatory cytokines, such as IFNγ, or toll-like receptor (TLR) ligands activate Mϕs into the proinflammatory M1 type with microbicidal and tumoricidal activity. Alternatively, Th2 cytokines IL-4/IL-13 promote M2 Mϕs with a tissue remodeling or immunoregulatory phenotype, while stimulation with IL-10, transforming growth factor-β (TGF-β), or glucocorticoids generates highly immunosuppressive “M2-like” Mϕs (3–5). The shift in the Mϕ polarizing stimuli during the resolution phase of infection is thought to convert M1 Mϕs to the M2 type, restoring tissue homeostasis (4, 6).

Recently, it has become apparent that ligation of TLRs or cytokine receptors also triggers profound changes in key metabolic events in Mϕs, enabling coordinate induction and maintenance of Mϕ effector activities (7–9). In M1 Mϕs, aerobic glycolysis is induced to readily provide cells with energy in a form of adenosine 5′-triphosphate (ATP). Aerobic glycolysis additionally feeds to the pentose phosphate pathway for production of nucleotides and NADPH, the latter being required for generation of microbicidal reactive oxygen species (8, 9). Furthermore, the glycolytic switch is associated with an increase in several metabolic intermediates that are incorporated into signaling pathways to support the inflammatory phenotype. In contrast, M2 Mϕs rely on oxidative metabolism that enables long-term cell survival and promotes M2 functions (7). Another example is l-arginine metabolism, which is a hallmark of differently polarized mouse Mϕs (10). In M1 Mϕs, arginine is a substrate to nitric oxide synthase (NOS2) induced by proinflammatory stimuli to produce antibacterial NO. In M2 Mϕs, arginine is metabolized by the M2 marker arginase 1 (Arg1) to urea and l-ornithine, a precursor of polyamines important for wound healing. Additionally, Arg1 action limits arginine availability to bystander proliferating T cells, leading to their suppression (10). While these key metabolic differences between M1 and M2 Mϕs are widely accepted, the metabolic cues that control the switch between different Mϕ phenotypes are not well understood.

Rheumatoid arthritis (RA) is an autoimmune disease characterized by chronic synovial inflammation and hyperplasia causing joint destruction (6, 11). Activated M1 Mϕs crucially contribute to disease pathology and their numbers in the sublining synovial layer predict severity of the disease (12). Interestingly, Mϕs expressing M2 markers, such as CD163 or folate receptor β (FRβ) were also identified in inflamed synovia (13–16). However, it is unclear whether and how these Mϕs with proposed anti-inflammatory properties contribute to disease pathology or whether they emerge to counteract inflammation. Hence, identifying and enhancing intrinsic pathways that would contribute to the resolution of inflammation in RA is an unmet need in RA therapy.

To elucidate mechanisms how different Mϕ subtypes contribute to chronic inflammation in RA and to identify pathways controlling their identity, we aimed to generate variously activated human GM-CSF- or M-CSF-differentiated Mϕs and address their ability to produce inflammatory mediators and influence T cell responses. We show that FRβ^+^ M-CSF-dependent Mϕs respond to proinflammatory stimuli by modulating gene expression of the purinergic pathway as a means to produce and respond to extracellular adenosine. Adenosine skews these cells toward the M2 state and suppresses autologous T cells. GM-CSF-dependent Mϕs resist this adenosine-mediated switch, so that only specific enhancement of the purinergic metabolism in the FRβ^+^ Mϕs potently limits inflammation in the arthritis mouse model.

## Materials and Methods

### Reagents

LPS (*Escherichia coli* serotype O55:B5) and adenosine were purchased from Sigma-Aldrich (St. Louis, MO, USA). Deuterated adenosine was from CDN Isotopes (Quebec, Canada). Adenosine 5′-triphosphate disodium salt (ATP) was from Thermo Fisher Scientific (Waltham, MA, USA). Recombinant human M-CSF, IFNγ, and IL-10 were obtained from Peprotech (Rocky Hill, NJ, USA). Recombinant human GM-CSF and IL-4 were from Novartis AG (Basel, Switzerland). The RPMI 1640 medium, l-glutamine, streptomycin, penicillin, and heat-inactivated fetal calf serum (FCS) were obtained from Gibco, Thermo Fisher Scientific. CD39 inhibitor POM-1 was from Tocris Bioscience (Bristol, UK). The cell proliferation dye CFSE and calcium sensor Fluo-4, AM was from Molecular Probes, Thermo Fisher Scientific. Brilliant Violet 421-conjugated streptavidin used as a second step in flow cytometry analyses was purchased from BioLegend (San Diego, CA, USA). Phorbol 12-myristate 13-acetate (PMA), ionomycin calcium salt (ionomycin) from *S. conglobatus* and monensin A sodium salt (monensin) were purchased from Sigma-Aldrich.

### Antibodies

The anti-FRβ monoclonal antibody (mAb) (clone EM-35) (17); was provided by EXBIO (Vestec, Czech Republic), either as purified or conjugated with Alexa Fluor 488, Alexa Fluor 647, or biotin. The second anti-FRβ mAb used in this study [clone 36b (18)] was purified using a Protein A Sepharose column and conjugated with phycoerythrin (PE) or biotin. EXBIO also provided Pacific Blue-conjugated CD14 mAb (clone MEM-18), FITC-conjugated CD64 mAb (clone 10.1), PerCP-conjugated CD86 mAb (clone BU63), Alexa Fluor 700-conjugated anti-MHC class II mAb (clone MEM-136 recognizing the β chain of HLA DR + DP), and allophycocyanin-conjugated CD4 mAb (clone MEM-241). Pacific Blue- and PE-conjugated CD69 mAb (clone FN50), FITC-conjugated mAbs to CD1a (clone HI149), CD8 (clone SK1), CD80 (clone 2D10), PE-conjugated mAb to CD73 (clone AD2) and to CD25 (clone BC96), PE-Cy7- and Brilliant Violet 421-conjugated CD39 mAb (clone A1), PerCP-conjugated mAb to CD16 (clone 3G8), PerCP-Cy5.5-conjugated mAbs to CD163 (clone GHI/61) and CD209 (clone 9E9A8) and allophycocyanin-Cy7-conjugated CD206 mAb (clone 15-2) were purchased from BioLegend. FITC-conjugated mAb to CD40 (clone LOB7/6) was from AbD Serotec (Oxford, UK). Allophycocyanin-conjugated mAb to CD25 (clone 4E3) was from Miltenyi Biotec (Bergisch Gladbach, Germany). For intracellular staining of T cells, the anti-FOXP3 mAb (clone 206D, conjugated to Alexa Fluor 647), FITC-conjugated anti-IFNγ mAb (clone 4S.B3), and PE-conjugated anti-IL-17A mAb (clone BL168) were purchased from BioLegend. The CD3 mAb OKT3 specific for the CD3ε chain was obtained from Centocor Ortho Biotech (Horsham, PA, USA). The mAbs L293 to CD28 and FITC-conjugated Leu4 to CD3 were purchased from BD Biosciences (Franklin Lakes, NJ, USA). mAbs to CD8 (clone MEM-87), CD14 (clone MEM-18), CD16 (clone MEM-154), CD19 (clone WIN19), CD20 (clone MEM-97), CD56 (clone MEM-188), used for CD4^+^ T cell isolation, and a CD147 mAb (clone MEM-M6/1) used in flow cytometry experiments were a kind gift of Vaclav Horejsi, Institute of Molecular Genetics, Academy of Sciences of the Czech Republic, Prague, Czech Republic. mAb to PD-L1 (clone 5-OM496) was a kind gift of Otto Majdic, Institute of Immunology, Medical University of Vienna, Vienna, Austria. Allophycocyanin-conjugated goat anti-mouse IgG + IgM Ab used as the second step in flow cytometry experiments was from Jackson ImmunoResearch Laboratories (West Grove, PA, USA). Additionally, Beriglobin P (CSL Behring, King of Prussia, PA, USA) was used for Fc receptor blockade in flow cytometry experiments.

For the experiments in the mouse, rat anti-murine-FRβ mAb was used (19), followed by a Dylight 488-conjugated anti-rat secondary Ab (eBioscience, Thermo Fisher Scientific, San Diego, CA, USA). Anti-murine PE-Cy7-conjugated CD11b mAb (clone M1/70) was from BD Biosciences. PerCP-Cy5.5-conjugated F4/80 mAb (clone BM8) and eFluor660-conjugated CD39 Ab (clone 24DMS1) were from eBioscience. Brilliant Violet 605-conjugated CD73 (clone T4/11.8) was from BioLegend.

### Cell Isolation and Culture

Human blood monocytes of healthy donors were isolated and differentiated to Mϕs and activated as previously described (20). Briefly, 7-day Mϕ differentiation was induced by 25 ng/ml GM-CSF or 50 ng/ml M-CSF; subsequent activation by 100 ng/ml LPS plus 25 ng/ml IFNγ, 20 ng/ml IL-4 or 20 ng/ml IL-10 for 48 h. When indicated, 20 µM POM-1, 100 µM ATP, 10 µM adenosine (or vehicle control) was added. CD4^+^ T cells were isolated from monocyte-depleted fraction by negative selection (21) and frozen. Synovial fluid cells were from knees of patients with inflammatory arthritis and analyzed immediately.

The study using human material was performed in accordance with the Declaration of Helsinki, informed consent was obtained from all participants and research was approved by the Ethics Committee of the Medical University of Vienna (2177/2013, 559/2005).

### T Cell Proliferation Assay

Activated human Mϕs, seeded in U-bottom 96-well plates (18,000 cells/well), were carefully washed and the autologous CD4^+^ T cells, labeled 12 h prior the assay with 1 µM CFSE, were added at the concentration of 90,000/well (control). In parallel, T cells were polyclonally stimulated with soluble CD3 mAb OKT3 (1 µg/ml). Controls included T cells cultured alone with or without soluble CD3 mAb, or cultured with plate-bound CD3 mAb OKT3 (1 µg/ml) plus soluble CD28 mAb L293 (0.5 µg/ml). In some experiments, IL-10-blocking mAb or isotype-matched control mAb (10 µg/ml) was included. Cocultures were performed in duplicates in RPMI 1640 medium supplemented with 2 mM l-glutamine, 100 µg/ml streptomycin, 100 U/ml penicillin, and 5% heat-inactivated FCS (all from Gibco, Thermo Fisher Scientific); fresh medium (including mAbs) was supplemented every 3 days. T cell proliferation was monitored on day 7 by assessing CFSE staining by flow cytometry. For quantification, T cells were analyzed using FlowJo (Tree Star, Ashland, OR, USA) and electronically sorted according to the CFSE peaks into gates that represented the respective generations (*i*) of proliferating T cells. Then, the cell count in the individual gates (*N_i_*) was determined. The percentage of dividing T cells (from the parental population), %D, was calculated according to the formula:

(1) $$\%D=\frac{\sum_{1}^{i}\frac{N_{i}}{2^{i}}}{\sum_{0}^{i}\frac{N_{i}}{2^{i}}}\times 100,$$

where *i* is the generation number as discriminated by the CFSE gating (starting with 0 for the undivided population) and *N_i_* is the number of events (cells) in generation *i*. The division index, DI, defined by the average number of cell divisions that parental population has undergone, was calculated according to the formula:

(2) $$DI=\frac{\sum_{0}^{i}i\times \frac{N_{i}}{2^{i}}}{\sum_{0}^{i}\frac{N_{i}}{2^{i}}}.$$

Both statistics parameters used here are explained in detail elsewhere (22).

### Flow Cytometry

Flow cytometry analysis of human Mϕs was performed as previously described (20).

For the analysis of T cell surface antigens, cells were washed with precooled staining buffer (PBS containing 1% BSA and 0.02% NaN_3_) and incubated on ice for 30 min with 4.8 mg/ml human IgG (Beriglobin P) to prevent nonspecific binding of the mAbs to Fc receptors. Then, antibody–fluorochrome conjugates with appropriate isotype controls were added. Cells were incubated for 30 min on ice and then washed with staining buffer. Samples were analyzed on an LSRII flow cytometer (BD Biosciences) and the data were further processed with the FlowJo software. Living single cells were gated according to their forward- and side-scatter characteristics and dead cells were excluded using DAPI or 7-aminoactinomycin D (Sigma-Aldrich). Cells were scored positively if they had a higher fluorescence than the cutoff of 0.5% of the isotype control mAbs. In graphs, geometric mean of fluorescence intensity corrected for background staining using matched isotype control mAb is shown.

For FOXP3 analysis, T cells were first stained using mAbs against the surface markers CD4 and CD25 as described above, then fixed, permeabilized, and stained for FOXP3 using the FOXP3 Fix/Perm Buffer Set according to the manufacturer’s protocol (BioLegend).

For analysis of intracellular cytokine production, T cells were restimulated on day 5 with 16.2 nM PMA and 1 µM ionomycin for 6 h with addition of 1 µM monensin for the last 4 h. The cells were fixed with 4% paraformaldehyde and permeabilized in 0.1% saponin (both from Sigma-Aldrich) in PBS. Before adding anti-IFNγ and anti-IL-17A mAbs, cells were blocked with 2.4 mg/ml human IgG (Beriglobin P) diluted in the intracellular staining buffer (5% FCS, 0.1% saponin in PBS) that was also used for washing.

### Gene Expression Analysis

Total RNA was extracted with the PureLink RNA Mini Kit (Ambion, Austin, TX, USA) or TRIzol reagent (Invitrogen, Carlsbad, CA, USA) supplemented with β-mercaptoethanol for RNAse inhibition. cDNA synthesis, quantitative PCR using GoTaq qPCR Master Mix (Promega, Madison, WI, USA) and analysis was performed as previously described (23). Primers are listed in Table S1 in Supplementary Material. *ACTB* was used as endogenous control unless stated otherwise and results are reported relative to values for one of the samples as specified in figure legends.

For microarray analysis, RNA was isolated from the different Mϕ subsets using PureLink RNA Mini Kit (Ambion). 500 ng of total RNA from each sample (in biological triplicates) was used for amplification, labeling, and hybridization using GeneChip Primeview Human Gene Expression Array and 3′IVT Express Kit Bundle (Affymetrix, Santa Clara, CA, USA). RMA was used for normalization of the dataset (24) and further analysis was done with limma (25).

The microarray data were submitted to the GEO repository (accession No. GSE61298).

**Figure 1:**
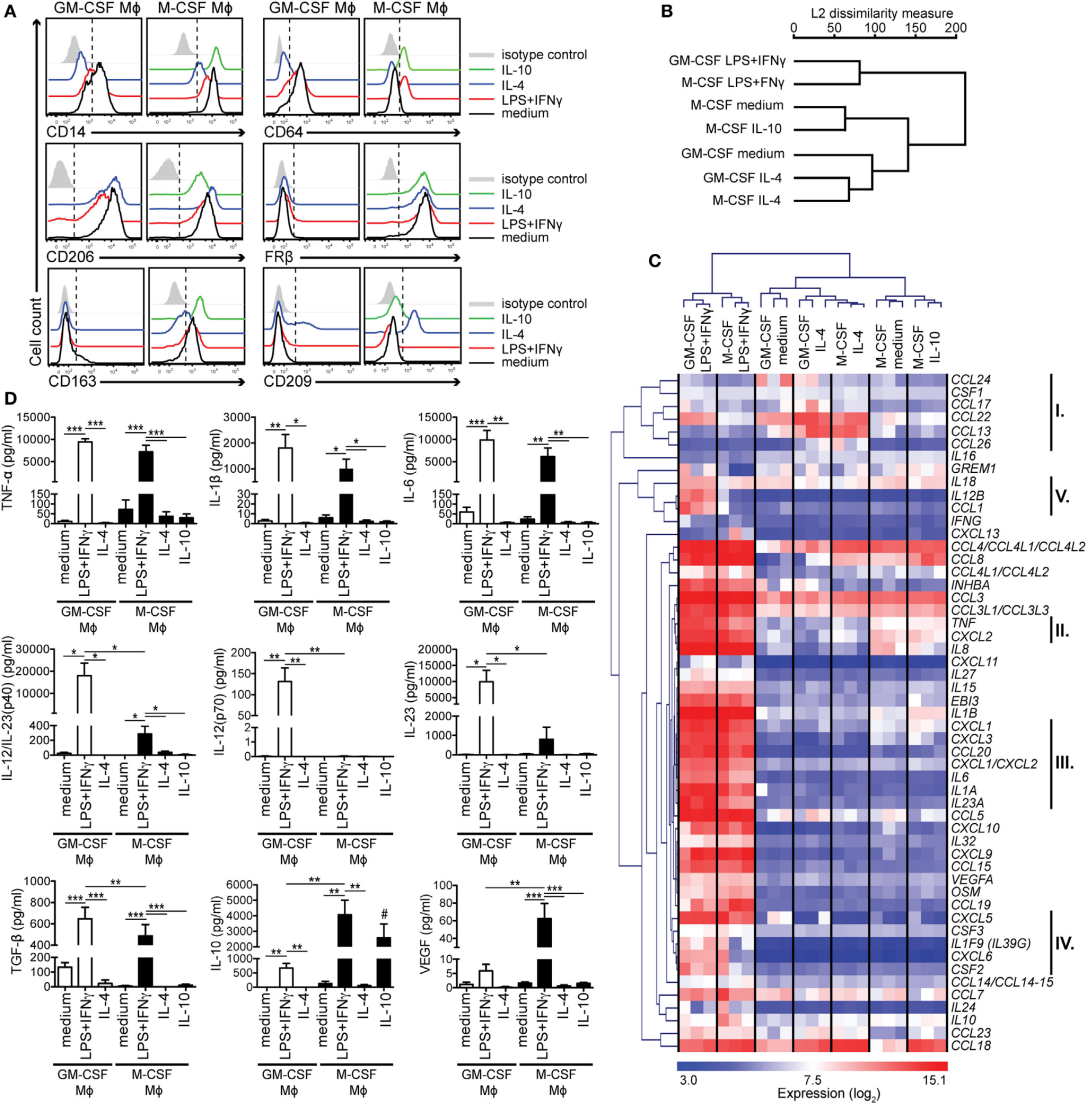
Activated human GM-CSF- and M-CSF-differentiated macrophage (Mϕ) subtypes are distinguishable by their surface marker, cytokine, and chemokine profiles. Human Mϕs were differentiated for 7 days with either GM-CSF or M-CSF and then activated for 2 days with the indicated stimuli. **(A)** Surface expression of Mϕ markers was measured by flow cytometry. Mock-activated cells are shown in black, cells activated with—LPS + IFNγ in red, with IL-4 in blue and with IL-10 in green. Control staining of mock-activated cells using the isotype control monoclonal antibodies is shown in gray (isotype control staining of activated cells was similar and is not depicted here). One representative of 10–12 experiments is shown. **(B)** Genome-wide analysis of Mϕ polarization was assessed by transcriptome profiling of Mϕ subtypes from **(A)**. Hierarchical clustering of Mϕ subtypes based on 22,067 gene probe sets (≈44.7%) that showed a significantly different expression (adj. *p* value <0.05) in at least one comparison. Data are combined from three independent experiments. **(C)** Heat map of transcripts of cytokine and chemokine genes based on the differentially expressed gene probe sets (115 gene probe sets; adj. *p* value < 0.05) with log_2_ expression > 7 in at least one sample. Important gene clusters discussed (I–V) are highlighted on the right. Mϕs have been generated from three donors and are visualized separately. **(D)** Active TGF-β from cell-free culture supernatants was determined using the *SERPINE1 (PAI-1)* promoter-driven luciferase reporter assay, while the other cytokines were measured by the Luminex analysis. The mean cytokine concentration ± SEM from 5 to 6 donors is shown. *p < 0.05, **p < 0.01, ***p < 0.001. Statistical significance was determined by one-way ANOVA with Tukey’s posttest. The hash key (#) indicates that the IL-10 levels in the IL-10-stimulated Mϕ subtype represent a mixture of released and residual IL-10 from the activation step and, therefore, were not evaluated.

Heat maps for specific genes were created using TM4 (26) using probe sets with expression above the threshold values (log_2_ > 5) in at least one sample. If a gene contained duplicate probe sets, their mean value was calculated and loaded into TM4. For Figure 1C, the threshold was increased to log_2_ = 7 and probe sets with differential expression upon activation or upon differentiation, respectively, were used (adj. *p* value < 0.05) and averaged before loading into TM4 to visualize differentially expressed genes with moderate to high expression only. Hierarchical clustering was done by TM4 based on Pearson correlation as the distance metrics.

### Cytokine Measurements

IL-1β, TNF-α, IL-6, IL-12 (p40, p70), IL-23, IL-10, vascular endothelial growth factor (VEGF), IL-2, IL-4, and IFNγ were measured from cell-free supernatants by Luminex analysis as detailed previously (23). Active TGF-β was determined using the *SERPINE1* (*PAI-1*) promoter-driven luciferase reporter assay (27).

### Extracellular ATP Degradation and Adenosine Measurements

Human Mϕ subtypes were differentiated and activated as described above. Mϕ-mediated degradation of 20 µM ATP in serum-free RPMI 1640 medium was monitored after 30 min at 37°C from cell-free supernatants using the Luminescent ATP detection Assay kit (Abcam, Cambridge, UK), omitting lysis step. For normalization, cells were lysed separately and the total protein amount was detected by Bradford protein assay (Bio-Rad, Hercules, CA, USA).

Adenosine was determined from the cell-free culture media of 7 days-differentiated and 2 days (48 h)-activated Mϕ cultures by liquid chromatography/mass spectrometry (LC/MS). For that, 100 µl of cell culture medium was spiked in with 1 µg deuterated adenosine as the internal standard and samples were deproteinated by chloroform–methanol extraction and lyophylized. The sample extract was dissolved in eluent (75% acetonitrile/100 mM aqueous ammonium acetate, pH 4.5) and 2 µl were injected onto the separation column (SeQuant^®^ ZIC^®^-cHILIC 100 mm × 0.3 mm, which was kindly provided by Merck, Darmstadt, Germany). Adenosine was separated using isocratic separation conditions on the Dionex nano RSLC HPLC (Thermo Fisher Scientific) system. The column was operated at 45°C using a flow rate of 8 µl/min. UV detection was performed at 260 nm prior to ESI-MS operated in positive ionization mode using a Bruker maXis Impact mass spectrometer (Bruker, Bremen, Germany). The MRM scan of the transition *m/z* 268.097 (MH+) to *m/z* 136.054 (MH+) was performed using the CID-MS/MS. The peak area of extracted ion chromatograms for *m/z* 268.097 were integrated using Data Analysis Version 4.1 (Bruker Daltonik, Bremen, Germany). The resulting peak area was corrected using the area of the internal standard with *m/z* 269.103 (MH+); MRM transition 269.103 to 137.053. Finally, adenosine concentration in the samples was calculated from the adenosine calibration curve ranging from 0 to 10 µg/ml cold adenosine spiked in with the internal standard and processed as described above.

### Live Cell Imaging

Human Mϕs differentiated with M-CSF for 7 days were activated with 100 ng/ml LPS plus 25 ng/ml IFNγ on low-adherent HydroCell plates (NUNC, Thermo Fisher Scientific) for 2 days, then detached using ice-cold 1.5 mM EDTA in HBSS (Gibco), washed with PBS supplemented with 10% FCS, and loaded with CD3 mAb OKT3 (50 µg/ml) for 30 min on ice. Then, Mϕs were stained with Brilliant Violet 421-labeled CD39 and PE-labeled CD73 mAbs (or labeled isotype-matched control mAbs) for 30 min on ice and washed twice with PBS supplemented with 10% FCS. One day prior to imaging, autologous CD4^+^ T cells were defrosted and left to recover overnight. In some experiments, on the day of imaging, living T cells were purified by gradient centrifugation using Lymphoprep (Axis-Shield, Oslo, Norway) and extensively washed with PBS. CD4^+^ T cells (10^6^/ml) were loaded with 1 µM Fluo-4 in complete culture medium for 30 min at 25°C and then washed twice with the imaging buffer [HBSS supplemented with 2% FCS, 10 mM HEPES pH 7.4 (Gibco), 1 mM MgCl_2_, and 1 mM CaCl_2_]. For imaging, first the Mϕs, then T cells were put onto a 1.0 borosilicate glass surface of an 8-well Lab-Tek II chamber slide (NUNC) at 25°C. Image acquisition was performed with a Leica DMI4000B microscope (Leica Microsystems; Wetzlar, Germany) equipped with a 40× immersion objective (Leica HCX PL Apo 40×, NA 1.25) and an Andor iXon Ultra-8871 EM-CCD camera (Andor Technologies; Belfast, UK) controlled by the Leica Application Suite Advanced Fluorescence software (version AF6000LX). Imaging of Fluo-4, PE and Brilliant Violet 421 excitation and emission light filtering was achieved through the Leica Quad-Sedat filter system (dichroic: 430,505,757,670) including two external filter wheels DFTC-Ex (350/50, 490/20, 555/26, 645/30) and DFTC-Em (455/50, 525/36, 605/52, 705/72). DIC and fluorescence images were collected at intervals of 1 min over 45–50 min. Synapse formation and T cell intracellular calcium dynamics determined by monitoring of the Fluo-4 fluorescence were analyzed with the open source image analysis software package Fiji (28).

### Human Serum Albumin (HSA) Coupling to methotrexate (MTX) and Folic Acid (FA)

*N*-(3-dimethylaminopropyl)-*N*-ethylcarbodiimide hydrochloride (EDAC) and *N*-hydroxysuccinimide (both from Sigma-Aldrich) were dissolved independently in dimethyl sulfoxide (DMSO) and mixed with MTX dissolved also in DMSO. The solution was kept under continuous stirring in a glass labware until complete dissolution. Activation was performed at 50°C in a water bath during 15 min. Activated MTX was then added drop by drop to HSA dissolved in 130 mM NaHCO_3_ buffer, pH 7.2 under continuous stirring at room temperature (RT) and the final solution incubated for a 20-min period at RT. Conjugation was followed by extensive dialysis against NaHCO_3_ buffer (membrane cut-off of 20 kDa) to eliminate free MTX. HSA and MTX concentrations were determined by measuring the optical density of the solution at 280 and 370 nm, respectively.

The same protocol was used to activate FA. Half of the HSA-MTX solution was then mixed with activated FA at RT under continuous stirring, followed by 20-min incubation at RT. The molecular ratio between HSA, MTX, and FA was calculated to be 1:1.6:1.2 by measuring absorbance of the different conjugates at 280 (for HSA) and 370 nm (for MTX and FA). All solutions were dialyzed against PBS and filter sterilized before use.

### Collagen-Induced Arthritis (CIA) Mouse Model and *Ex Vivo* Analysis of Mϕs by Flow Cytometry

6-week-old DBA/1JRj male mice were purchased from Janvier Laboratory (Le Genest-St.-Isle, France). Arthritis was induced with type II bovine collagen (CII; MD Bioscience, Zurich, Switzerland) as previously described in Ref. (29). Briefly, mice were injected intradermally at the base of the tail with 100 µg of CII emulsified in Freund’s adjuvant (BD DIFCO, Thermo Fisher Scientific). On day 21, mice were boosted with an intradermal injection of CII in incomplete Freund’s adjuvant (BD DIFCO, Thermo Fisher Scientific). Mice were monitored for evidence of arthritis in paws using a blind procedure. For each mouse, clinical severity of arthritis was scored (0-normal; 1-erythema; 2-swelling; 3-deformity; 4-necrosis) in 10 joints or group of joints (toes, tarsus, ankle of the hindleg and fingers, and carpus of the foreleg) as detailed elsewhere (29). The MTX (35 mg/kg; the HSA conjugates were used at the amount equivalent to 7 mg/kg of free MTX) and the vehicle control (PBS) treatments injected were randomized in each cage to avoid cage dependence of the clinical score. Treatment started 14 days after immunization, with intraperitoneal injections twice a week, and mice were scored the same day.

For flow cytometry analysis, ankles were dissected, digested with collagenase (50 µg/ml) diluted in RPMI 1640 medium without serum for 2 h at 37°C. Then, ankles were dissociated and the cell suspension was filtered through a 40 µm cell strainer. For surface staining, Fc receptors were blocked with normal goat and rabbit IgG (10 µg/ml) in PBS with 1% BSA for 30 min on ice. Cells were then stained in the same medium with an anti-murine FRβ for 30 min on ice, washed with PBS with 1% BSA and incubated with a Dylight 488-conjugated anti-rat secondary Ab (eBioscience) for 30 min on ice. After two consecutive washes, cells were stained with rat anti-mouse-CD11b, F4/80, CD39, and CD73 for 30 min on ice, washed again, and measured using an LSR II flow cytometer (BD Biosciences) equipped with the FACSDiva software; and the data were further processed with the FlowJo software.

The experiments using mouse models were approved by the French Ministry of Research and the Paris Descartes University Ethical Committee (CEEA N°34); agreement N°CEEA34.GB.029.11. All methods and experiments were performed in accordance with the relevant guidelines and regulations.

### Statistics

The number of independent experiments (human donors) and mice in animal studies is specified in the figure legends. The statistical significance between subsets of the particular Mϕ lineages was assessed as specified in the figure legends using Prism 5 (GraphPad Software, La Jolla, CA, USA). Additionally, for assessment of the difference between the samples activated with the same stimuli two-tailed unpaired *t*-test was used. In all analyses, statistical significance was accepted at *p* < 0.05.

## Results

### M-CSF-Dependent Mϕs Show a Skewed M1/M2 Profile in Response to M1 Activation Stimuli

To establish a model system for RA-associated Mϕs, we differentiated human CD14^hi^ monocytes into Mϕs by culturing them for 7 days with either GM-CSF or M-CSF, which are both upregulated in RA tissues (12). Mϕs were then treated with LPS + IFNγ for 48 h to mimic chronic inflammatory conditions (M1 state). Alternatively, we used IL-4 or IL-10 (the latter for the M-CSF-primed Mϕs only) to activate Mϕs to the M2/M2-like states (20). We confirmed the activation states by assessing surface expression of several Mϕ markers by flow cytometry (Figure 1A; Figure S1 in Supplementary Material that can be found with all other Supplementary Figures in Supplementary Material). GM-CSF-differentiated Mϕs were characterized by lower expression of the LPS coreceptor CD14 than M-CSF-differentiated Mϕs, while CD64 was strongly expressed by mock-activated GM-CSF-differentiated Mϕs. Both markers were highly expressed by LPS + IFNγ- and IL-10-stimulated Mϕs and strongly downregulated in response to IL-4. The commonly used M2 marker CD206 (5), was not found specific for M2 Mϕs, since it was highly expressed by all subtypes, though further upregulated by IL-4. In contrast, the M2 marker FRβ (3) was expressed at high levels exclusively by the M-CSF lineage regardless of subsequent polarization. Other M2 markers CD163 and CD209 were also preferentially expressed on M-CSF-differentiated Mϕs, but in contrast to FRβ, expression of these markers varied, depending on the activation stimulus: CD163 expression was the highest in the presence of IL-10, while CD209 was upregulated in response to IL-4 in both lineages. Thus, by probing for these Mϕ markers, we are able to discriminate GM-CSF- versus M-CSF-differentiated Mϕs and assess their activation status. These data also reveal that M-CSF-dependent Mϕs express certain M2 markers even in the proinflammatory environment.

To gain a better insight into Mϕ polarization, we analyzed the transcriptome of these seven subtypes using a whole-human genome microarray. On the transcriptome-wide level, Mϕs clus-tered according to the activation regime, with LPS + IFNγ-activated Mϕs of both lineages in one branch and IL-4-activated Mϕs segregating at the furthest end of the other branch (Figure 1B). Similar results we obtained when we focused onto analysis of genes encoding cytokines and chemokines, which are both crucial effector molecules and markers of different Mϕ types (Figure 1C). IL-4-activated Mϕs of both lineages highly expressed a cluster of M2-associated chemokine genes (cluster I; *CCL13, CCL17, CCL22, CCL24, CCL26*). Again, the most pronounced changes were caused by LPS + IFNγ stimulation. Here, we detected a robust upregulation of genes encoding proinflammatory cytokines and chemokines. Nevertheless, a subgroup of them (clusters II–IV) was expressed at lower levels in the M-CSF-differentiated LPS + IFNγ subtype: while the genes in cluster II (*TNF* and *CXCL2*) and III (*IL1A, IL6, IL23A, CXCL1*-*CXCL3*, and *CCL20*) were found to be uniformly upregulated to some extent, several genes from cluster IV (*CSF2, CXCL6*, and *IL1F9*) were mildly upregulated only in one donor. Furthermore, this Mϕ subtype barely upregulated *IL12B*, encoding the p40 subunit of IL-12/IL-23, *IL18*, and *CCL1* (cluster V), but expressed *IL10*.

To confirm the transcriptome data, we measured the cytokines in the culture supernatants of the activated Mϕs (Figure 1D). LPS + IFNγ-stimulated Mϕs of both lineages released high amounts of proinflammatory cytokines TNF-α, IL-1β, and IL-6 and immunoregulatory TGF-β. These cytokines were slightly better produced by the GM-CSF-dependent subtype. However, the two cell types showed clear differences in secretion of the Th1- and Th17-instructing cytokines IL-12 and IL-23: GM-CSF-differentiated Mϕs scored highly positive, while those differentiated by M-CSF did not; instead, they produced high levels of IL-10 and VEGF.

Taken together, these results reveal a distinct response of GM-CSF- and M-CSF-differentiated Mϕs to LPS + IFNγ stimulation that is not apparent on transcriptome-wide level, and indicate that M-CSF-differentiated/LPS + IFNγ-stimulated Mϕs with the M1/M2 profile might be less inflammatory.

### M1-Stimulated M-CSF-Dependent Mϕs Inhibit T Cell Responses

**Figure 2:**
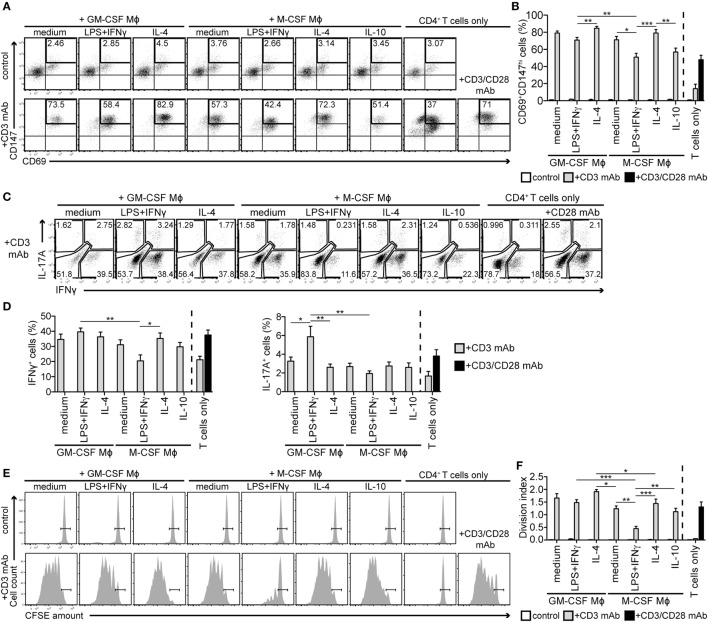
M-CSF-differentiated macrophages (Mϕs) activated with LPS + IFNγ suppress activation, cytokine production, and proliferation of autologous CD4^+^ T cells. Mϕs described in Figure 1 were cocultured with CFSE-labeled autologous CD4^+^ T cells in the presence or absence of the stimulating CD3 monoclonal antibody (mAb) OKT3. As a control, T cells were cultured alone as indicated. **(A,B)** CD4^+^ T cells were stained for activation markers CD69 and CD147 after 2 days coculture with different Mϕ subtypes. The percentage of activated cells is given in the CD69^+^CD147^hi^ gate. One representative experiment **(A)** and statistics **(B)** of eight independent experiments is shown. **(C,D)** Mϕ subtypes were cocultured with unlabeled autologous CD4^+^ T cells in the presence of the CD3 mAb. After 5 days of coculture, T cells were restimulated with phorbol 12-myristate 13-acetate and ionomycin for 6 h and stained for IFNγ and IL-17A. One representative experiment **(C)** and the mean percentages (representing the sum of the respective single and double-positive gates) ± SEM **(D)** of the IFNγ- and IL-17A-expressing T cells of seven independent experiments are shown. **(E)** Proliferation of CFSE-labeled T cells cocultured with autologous Mϕs ± CD3 mAb was determined by CFSE dilution on day 7 using flow cytometry. One representative experiment, where CFSE^hi^ non-dividing cells are gated, of nine independent experiments is shown. **(F)** T cell division index was calculated according to the CFSE peaks. Data represent means ± SEM of nine independent experiments. Expression of activation markers **(B)**, cytokines **(D)**, and proliferation **(F)** of the CD3-stimulated T cells in Mϕ cocultures were statistically evaluated by one-way ANOVA with Tukey’s posttest; *p < 0.05, **p < 0.01, ***p < 0.001.

Next, we probed for Mϕ ability to stimulate T cells. In coculture experiments with CFSE-labeled autologous CD4^+^ T cells, no Mϕ subtype activated T cells without ectopic T cell antigen receptor (TCR) triggering, as revealed by co-staining of the T cell activation markers CD69 and CD147 on day 2. But in the presence of soluble CD3 mAb, all subtypes were able to provide T cells with the necessary second stimulus (Figure 2A). Yet, the degree of T cell activation varied, with the lowest percentage of activated (CD69^+^CD147^hi^) T cells when cocultivated with the M-CSF-differentiated/LPS + IFNγ-stimulated Mϕs (Figures 2A,B). We found that this was due to the impaired upregulation of the late activation marker CD147 (30), while robustly upregulated CD69 (an early activation marker) and CD25 (an intermediate activation marker) (31) were not significantly affected (Figure S2A in Supplementary Material). T cells cocultured with M-CSF-differentiated/LPS + IFNγ-stimulated Mϕs also released lower amounts of IL-2, IFNγ, IL-4, and IL-10 compared to the other samples (Figure S2B in Supplementary Material). Hyporeactivity of these T cells was also detectable, when proximal TCR signaling was bypassed by restimulation with the mitogen PMA and the calcium ionophore ionomycin, as revealed by intracellular staining of IFNγ and IL-17A on day 5 (Figures 2C,D). In contrast, when cocultured with similarly activated GM-CSF-differentiated Mϕs, T cells released high levels of all cytokines measured (Figure S2B in Supplementary Material) and contained the highest frequency of IFNγ- and IL-17A-producing cells (Figures 2C,D).

Finally, we monitored T cell proliferation by quantitating CFSE dilution 7 days after coculture. As shown in Figure 2E, no Mϕ subtype was able to markedly induce T cell proliferation under control conditions. CD3 mAb-stimulated T cells cultured alone were found anergic, likely due to missing CD28-mediated co-stimulation, while high T cell proliferation was detected in cocultures with all types of GM-CSF-differentiated Mϕs and M-CSF-differentiated/IL-4-activated Mϕs (Figures 2E,F). In contrast, T cells cocultured with M-CSF-differentiated/LPS + IFNγ-activated Mϕs proliferated markedly less (Figures 2E,F). A time-course analysis showed that they practically stopped dividing between days 5 and 7 (Figure S2C in Supplementary Material). Based on these data, we speculated that a combination of M-CSF and proinflammatory stimuli drives Mϕs toward the immunosuppressive phenotype.

### Mechanisms of T Cell Suppression Mediated by the Immunoregulatory Mϕ Subtype

To uncover the mechanism underlying this immunosuppressive phenotype, we tested induction of FOXP3^+^CD4^+^CD25^+^ regulatory T cells (Tregs). In control cocultures, the FOXP3^+^ cells were probably natural Tregs. As reported (32), FOXP3 expression moderately increased upon T cell activation. Lowest levels were detected in coculture with M-CSF-differentiated/LPS + IFNγ-stimulated Mϕs, revealing that they did not induce Treg differentiation (Figures S3A,B in Supplementary Material).

We, therefore, searched for cell-intrinsic mechanisms of immunoregulation. First, we checked antigen-presenting and costimulatory molecules. In agreement with the results of coculture assays, the potent stimulators (GM-CSF-differentiated Mϕs activated with LPS + IFNγ or IL-4) expressed highest levels of MHC class II and the costimulatory receptors CD80, CD86, CD40 and, in some donors, also the (glyco)lipid-presenting molecule CD1a. The immunosuppressive M-CSF-differentiated/LPS + IFNγ-stimulated Mϕs expressed high levels of MHCII, CD80, CD40, but not CD86 (Figures S3C,D in Supplementary Material). Nevertheless, since other M-CSF-differentiated T cell-stimulating subtypes expressed even lower levels of costimulatory molecules (Figures S3C,D in Supplementary Material) and M-CSF-differentiated/LPS + IFNγ-activated Mϕs suppressed T cell proliferation even in the presence of CD3 + CD28 mAbs (Figures S2D,E in Supplementary Material), we excluded missing co-stimulation as a possible mechanism.

Second, based on our microarray data we compared expression of genes, which were differentially regulated in response to LPS + IFNγ in GM-CSF- versus M-CSF-differentiated Mϕs. We focused on genes involved in T cell stimulation or inhibition (33). This analysis corroborated preferential upregulation of genes encoding proinflammatory cytokines (*IL12B, TNF, IL6*) and costimulatory molecules (red highlighted in Figure S3E in Supplementary Material) by GM-CSF-primed Mϕs, while both Mϕ lineages upregulated several genes associated with T cell inhibition or immunoregulation: *IL10, CD274* (*PD-L1*), *PDCD1LG2* (*PD-L2*), *IDO1, IDO2, IL2RA* (*CD25*), and *SOCS1*-*SOCS3* (blue and green highlighted in Figure S3E in Supplementary Material). In subsequent staining experiments, we confirmed that PD-L1, which inhibits T cells by engaging PD-1 (33), and CD25, which scavenges IL-2 from T cells (34), were uniquely upregulated by LPS + IFNγ (Figure S3F in Supplementary Material). Since their surface expression varied minimally between the T cell-stimulating and T cell-inhibitory subset, they were unlikely to mediate T cell suppression. Similarly, we also excluded IL-10 as a sole mediator of the suppressor phenotype, as the blocking IL-10 mAb did not restore T cell proliferation in coculture experiments (data not shown).

### Mϕs Alter Extracellular Purine Metabolism in Response to Proinflammatory Stimuli

**Figure 3:**
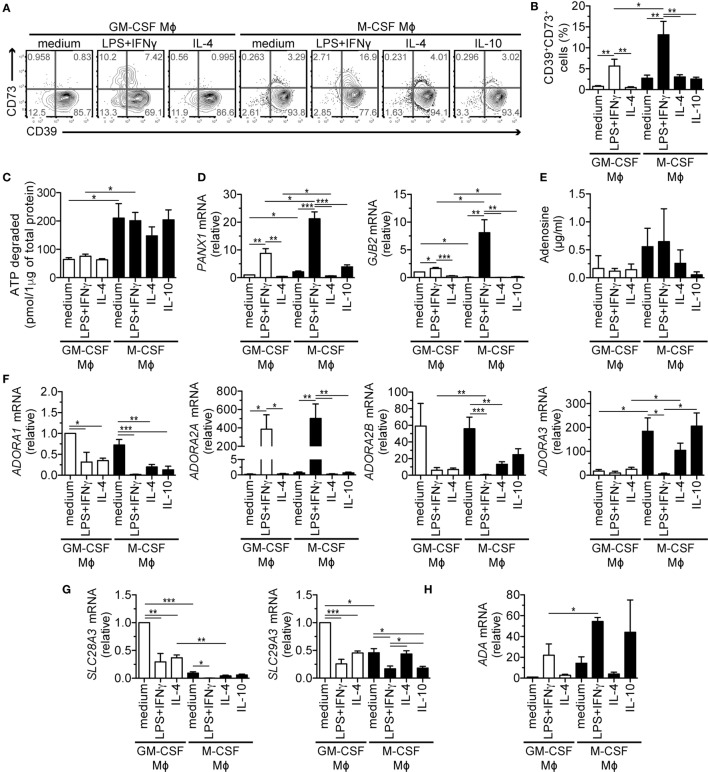
Expression and function of genes of the purinergic pathway upon LPS + IFNγ treatment of M-CSF- or GM-CSF-differentiated macrophages (Mϕs). Human Mϕ subtypes were prepared as described in Figure 1. **(A,B)** Surface expression of AMP- and adenosine-producing enzymes CD39 and CD73 on different Mϕ subsets was determined by flow cytometry. One representative experiment **(A)** and the mean percentages (±SEM) of CD39^+^CD73^+^ cells from five experiments are shown **(B)**. **(C)** Degradation of 20 µM ATP by Mϕ subtypes. After 30 min, remaining ATP was measured and normalized to the Mϕ protein content. **(D)** mRNA expression of genes encoding ATP-releasing channels pannexin 1 (*PANX1*) and connexin 26 (*GJB2*) was analyzed by qRT-PCR. **(E)** Adenosine was measured in the cell-free culture supernatants by mass spectrometry. **(F–H)** mRNA expression of genes encoding **(F)** adenosine receptors (*ADORA1-3*), **(G)** adenosine uptake channels (*SLC28A3, SLC29A3*) and **(H)** the adenosine-catabolizing enzyme adenosine deaminase (*ADA*) was analyzed by qRT-PCR. To compare the expression of the various adenosine receptors, we normalized the data to *ADORA1* mRNA levels in GM-CSF control Mϕs that were set to one. Other genes were normalized to the respective levels found in GM-CSF control Mϕs that were set to one **(D,G,H)**. Data in **(C–H)** represent mean values ± SEM of three **(C,D,G,H)** to five **(E,F)** donors. Statistical significance was assessed by one-way ANOVA with Tukey’s posttest; *p < 0.05, **p < 0.01, ***p < 0.001.

Our search for LPS + IFNγ-regulated genes involved in T cell suppression revealed altered expression of several genes involved in adenosine metabolism and signaling (*NT5E*, coding for the ecto-5′-nucleotidase CD73, and adenosine receptors *ADORA2A, ADORA2B*; Figure S3E in Supplementary Material). Extracellular adenosine generated from ATP released by activated T cells was found to potently suppress T cell functions (34, 35). The canonical pathway responsible for conversion of extracellular ATP to adenosine (Figure S4A in Supplementary Material) is represented by ectonucleotidases CD39 (ENTPD1) and CD73 (36, 37). We, therefore, measured surface expression of these enzymes on Mϕs by flow cytometry. All types of M-CSF-differentiated Mϕs expressed high levels of surface CD39; GM-CSF-differentiated Mϕs scored also positively but to a lesser extent (Figures 3A,B) and exhibited a lower capacity to degrade exogenous ATP (Figure 3C). CD73 was specifically upregulated in LPS + IFNγ-activated Mϕs secreting high amounts of TGF-β, IL-1β, and TNF-α (Figure 1D), which were shown to induce CD73 in non-Mϕ cells (38, 39). Remarkably, on the surface of the immunostimulatory GM-CSF-differentiated Mϕs, CD39 and CD73 appeared in two different subpopulations in a mutually exclusive manner. In contrast, M-CSF-differentiated Mϕs contained a prominent CD39^+^CD73^+^ double-positive subpopulation (Figures 3A,B).

Cohen et al. reported that mouse Mϕs release ATP in response to TLR stimulation through pannexin-1 (Panx1) channels (40). In line with these data, we detected profound upregulation of the *PANX1* and *GJB2* transcripts, which code for the ATP-releasing channels Panx1 and connexin-26 in the immunosuppressive Mϕs (Figure 3D). Other ATP-releasing channels, P2X and P2Y nucleotide receptors were expressed similarly by several subtypes (Figure S4B in Supplementary Material). To test whether M-CSF- and LPS + IFNγ-stimulated Mϕs indeed produce adenosine in a Panx1/connexin-26/CD39/CD73-dependent manner, we measured extracellular adenosine in Mϕ culture media. We detected the highest levels of adenosine in supernatants of M-CSF-differentiated Mϕs activated with the M1 stimuli, but these differences were not significant (Figure 3E). Therefore, we assessed the expression of adenosine-binding and -degrading proteins (Figures 3F–H; Figure S4B in Supplementary Material). Adenosine receptor transcripts, encoded by the *ADORA* genes, intensely fluctuated (Figure 3F): *ADORA1* mRNA levels were minimal and together with *ADORA2B* transcripts, they decreased upon Mϕ activation, while *ADORA3* was predominantly expressed in the M-CSF lineage with exception of the LPS + IFNγ-stimulated subset. The most prominent change was detected in *ADORA2A* transcripts upon LPS + IFNγ treatment that increased most in the immunosuppressive M-CSF-differentiated subtype. Notably, this subtype expressed the lowest amounts of other adenosine receptors and adenosine reuptake channels *SLC28A3* and *SLC29A3* (Figure 3G); however, it expressed high levels of adenosine-catabolizing adenosine deaminase (Figure 3H). Taken together, these data indicate that proinflammatory stimuli affect gene expression of the purinergic pathway in Mϕs. Further, they suggest that extracellular adenosine production and signaling is more efficient in the M-CSF than in the GM-CSF lineage.

### The Mϕ Purinergic Pathway Dynamically Responds to Changes in Extracellular Nucleotides

**Figure 4:**
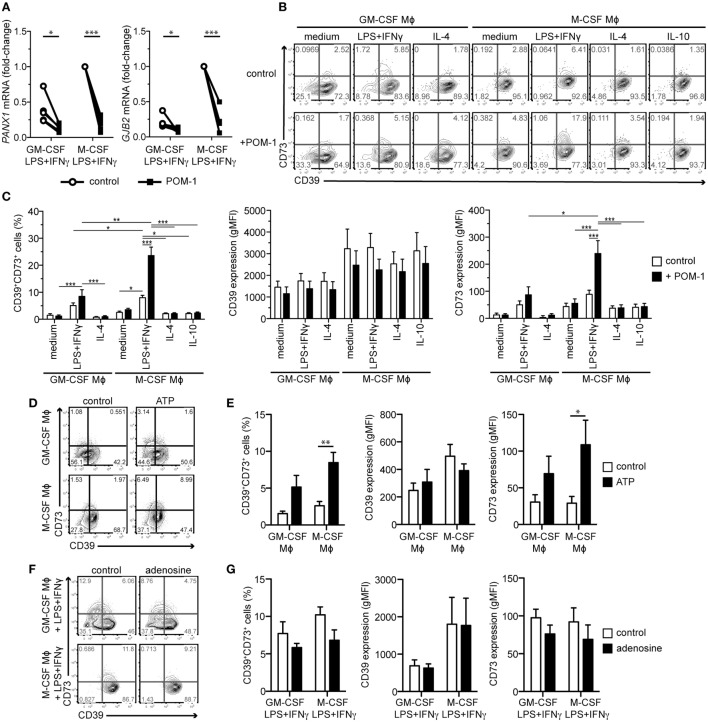
Effects of the CD39 inhibitor POM-1, extracellular ATP, and extracellular adenosine on expression of ATP release channels and adenosine-producing enzymes. **(A)** Macrophages (Mϕs) of both lineages were activated for 2 days with LPS + IFNγ in the absence (control) or presence of 20 µM POM-1. Expression of the genes encoding ATP release channels *PANX1* and *GJB2* was then analyzed by qRT-PCR. To compare gene expression, the data were normalized to the mRNA levels in the M-CSF LPS + IFNγ-stimulated Mϕs that were set to one. Data represent five different donors. **(B–G)** Mϕs were differentiated and activated as indicated. POM-1, ATP, adenosine, or respective controls were added during the activation step. **(B)** The expression of CD39 and CD73 on the indicated Mϕ subtypes in the absence or presence of 20 µM POM-1 was analyzed by flow cytometry. **(C)** Quantification of the data shown in **(B)** (*n* = 12). **(D,E)** Expression of CD39 and CD73 on GM-CSF or M-CSF-differentiated Mϕs in the absence or presence of 100 µM ATP was analyzed by flow cytometry. One representative experiment **(D)** and statistics from four independent experiments **(E)** is given. **(F,G)** Flow cytometry analysis of CD39 and CD73 expression on the surface of LPS + IFNγ-stimulated GM-CSF- or M-CSF-differentiated Mϕs in the absence or presence of 10 µM adenosine. One representative experiment **(F)** and statistics from four independent experiments **(G)** is given. Statistical significance was assessed by unpaired two-tailed *t*-test or one sample *t*-test for GM-CSF- or M-CSF-differentiated Mϕs, respectively **(A)** or by two-way ANOVA with Bonferroni posttest **(C,E,G)**; *p < 0.05, **p < 0.01, ***p < 0.001.

In order to assess the functionality of the purinergic pathway in LPS + IFNγ-stimulated Mϕs, we blocked the prime enzyme CD39 with the small molecule inhibitor POM-1 during the 2-day activation step. We observed that the CD39 blockade suppressed the LPS + IFNγ-upregulated expression of *PANX1* and *GJB2* transcripts, encoding the ATP-releasing channels (Figure 4A). The POM-1 treatment further increased the LPS + IFNγ-induced expression of CD73 in the T cell activating (GM-CSF-differentiated) and especially in the immunosuppressive (M-CSF-differentiated) subtype, enhancing the CD39^+^CD73^+^ subpopulation, while no such effect was observed in the other Mϕ subtypes (Figures 4B,C). POM-1-mediated upregulation of CD73 in LPS + IFNγ-treated Mϕs was likely caused by accumulated extracellular ATP resulting from CD39 inhibition, because exogenously added ATP was also able to increase CD73 expression, although significance was reached only in the M-CSF-dependent subset (Figures 4D,E). Based on these results, we speculated that CD73 expression could be also modulated by extracellular adenosine in a negative feedback loop. Indeed, we observed a trend toward the reduction of CD73 expression on the surface of both LPS + IFNγ-stimulated Mϕs that were treated exogenously with adenosine (Figures 4F,G). Taken together, these data suggest that the expression of several genes of the purinergic pathway in Mϕs is modulated by extracellular ATP and its degradation products to ensure optimal functionality of the pathway.

### Mϕs Enrich CD39 Within the Immunological Synapse to Attenuate T Cell Activation

**Figure 5:**
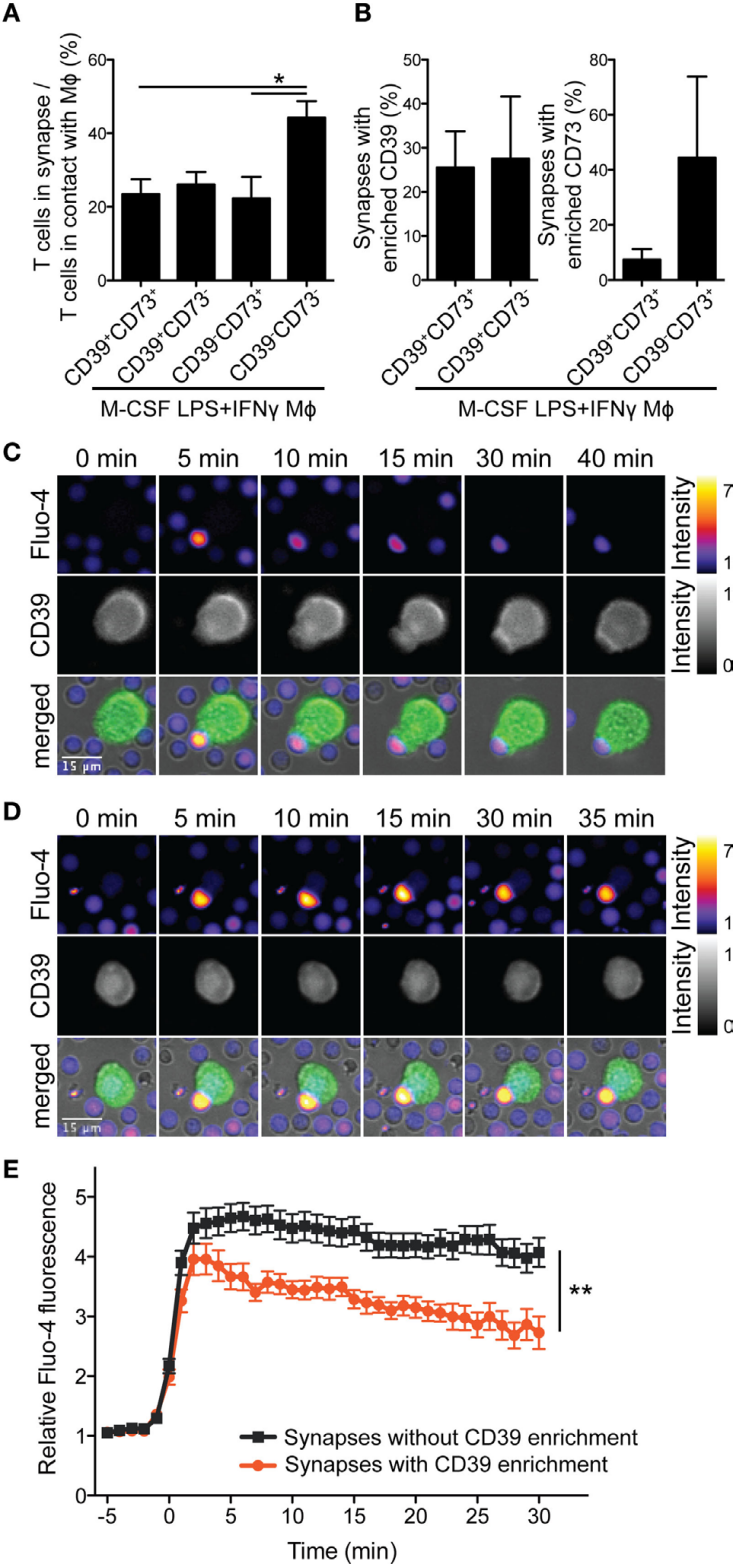
CD39 on M-CSF-differentiated and LPS + IFNγ-activated macrophages (Mϕs) influences the frequency of the synapse formation and the quality of the calcium response of engaged CD4^+^ T cells. Human Mϕs were differentiated for 7 days with M-CSF and then activated for 2 days with LPS + IFNγ. Afterward, they were loaded with CD3 monoclonal antibody (mAb), stained with PE-conjugated CD73- and Brilliant Violet 421-conjugated CD39 mAbs, and incubated with Fluo-4-loaded autologous CD4^+^ T cells. Time-lapse microscopy images were acquired every minute for 45–55 min. **(A)** The average numbers of T cells engaged in the synapse and T cells that touched CD39^+^CD73^+^, CD39^+^CD73^−^, CD39^−^CD73^+^, or CD39^−^CD73^−^ Mϕs were counted. Data are mean ± SEM of three experiments. Statistical significance was assessed by one-way ANOVA with Tukey’s posttest; *p < 0.05. **(B)** Mean percentage of Mϕ-T cell conjugates, where Mϕ CD39 or CD73 were enriched in the immunological synapse. **(C,D)** Montage of synapse formation between a CD39^+^CD73^−^ Mϕ and a Fluo-4-labeled autologous CD4^+^ T cell, where Mϕ CD39 was recruited to the immunological synapse **(C)** or not **(D)**. Time was counted from the initial contact between the T cell and the Mϕ. In overlays, Fluo-4 (in pseudocolor; range is depicted on the right), CD39 (in green) and brightfield pictures are shown. **(E)** Quantification of mean calcium signals of engaged Fluo-4-loaded CD4^+^ T cells from **(C,D)** over time. Data represent mean values ± SEM of 20 or 45 cells, respectively, calcium flux of which was traceable for >22 min. To statistically compare the traces, the area under curve (for fluxes 0–22 min) was calculated for each cell and groups were compared by unpaired two-tailed *t*-test; **p < 0.01.

In response to TCR stimulation, T cells release ATP into the immunological synapse that significantly contributes to localized calcium entry by the P2X1 and P2X4 ATP-gated calcium channels and T cell activation (41). To scrutinize whether T cell-derived ATP, converted to adenosine by Mϕ ectonucleotidases, contributes to immunosupression, we analyzed the first minutes of the CD3 mAb-mediated interaction between M-CSF-differentiated/LPS + IFNγ-activated Mϕs and CD4^+^ T cells by live cell video microscopy. CD39^+^CD73^+^, CD39^+^CD73^−^ and the seldomly observed CD39^−^CD73^+^ Mϕs exhibited lower capacity to engage T cells into an active immunological synapse than their CD39^−^CD73^−^counterparts (Figure 5A). Nevertheless, early T cell signaling was not blocked by the CD39^+^CD73^+^ Mϕs, since T cells increased intracellular calcium when they eventually got engaged. This prompted us to analyze the expression of the adenosine receptors in T cells. In line with the published data (42, 43), we found that the *ADORA2A* and *ADORA2B* transcripts gradually increased during T cell activation until day 3 (Figure S5 in Supplementary Material).

Of note, we observed that in ≈26% synapses of CD39^+^ Mϕs, CD39 staining was more intense than in areas outside of the synapse (Figures 5B,C), while CD73 was rarely enriched in these synapses. Furthermore, the recruitment of Mϕ CD39 into the immunological synapse was associated with a more rapid decrease of intracellular calcium flux in engaged T cells (Figures 5C–E; Movies S1 and S2 in Supplementary Material). These observations point to an essential role of Mϕ CD39 in modulating early phases of T cell activation. The CD39/CD73-generated adenosine did not seem to be crucial at this stage, possibly due to the low expression of adenosine receptors on resting and recently activated T cells (Figure S5 in Supplementary Material).

### CD39 Blockade Abrogates Mϕ Immunosuppressive Functions

**Figure 6:**
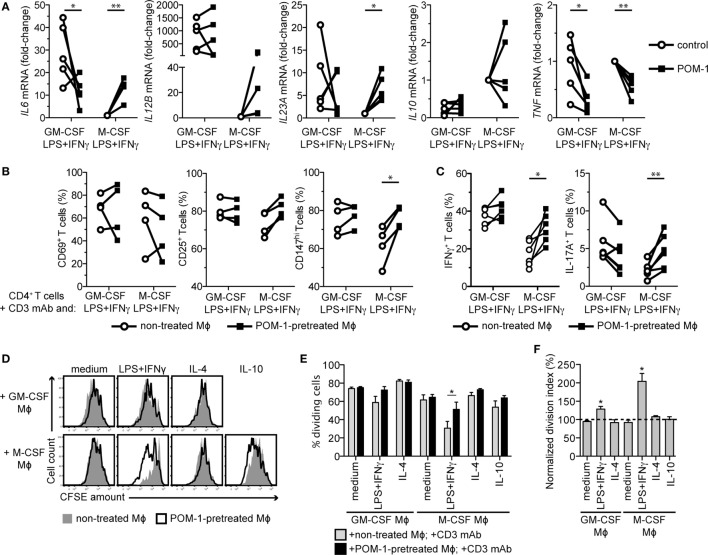
CD39 blockade by POM-1 mitigates the immunosuppressive phenotype of M-CSF-dependent LPS + IFNγ-activated macrophages (Mϕs). **(A)** Human Mϕs were differentiated for 7 days with GM-CSF or M-CSF and then activated for 2 days with LPS + IFNγ in the absence (control) or presence of 20 µM POM-1. Expression of the cytokine genes was then analyzed by qRT-PCR. To compare gene expression, the data were normalized to the mRNA levels in the M-CSF LPS + IFNγ-stimulated Mϕs that were set to one. Data represent five different donors. Statistical significance was assessed by unpaired two-tailed *t*-test or one sample *t*-test in case of M-CSF Mϕs. **(B)** Mϕs from **(A)** were cocultured with autologous CD4^+^ T cells in the presence of the CD3 monoclonal antibody (mAb). After 2 days, T cells were stained for the activation markers CD69, CD25, and CD147 and analyzed by flow cytometry. Percentages ± SEM from activation marker-positive T cells from four experiments is given. **(C)** On day 5 of coculture, T cells were restimulated with phorbol 12-myristate 13-acetate and ionomycin for 6 h and stained for IFNγ and IL-17A. Percentages ± SEM of the IFNγ- and IL-17A-expressing T cells of six independent experiments are shown. **(D)** Mϕs were differentiated for 7 days, activated for 2 days as indicated in the absence (control) or presence of 20 µM POM-1 and then cocultured with CFSE-labeled autologous CD4^+^ T cells stimulated (or not) with the CD3 mAb. Proliferation of T cells cocultured with non-treated Mϕs (filled gray histograms) or with the POM-1-pretreated Mϕs (open black histograms), respectively, was determined by quantifying the CFSE dilution after 7 days using flow cytometry. One representative of four experiments is shown. POM-1 pretreatment had no effect onto negligible proliferation of T cells in control cocultures without the CD3 mAb, thus these data are not shown. **(E)** The percentage of dividing T cells (from the parental population) cocultured with control or POM-1-pretreated Mϕs was calculated according to the CFSE peaks. **(F)** To determine the contribution of the CD39 blockade to the T cell proliferation, the division index of T cells cocultured with POM-1-pretreated Mϕs was normalized to the division index of respective T cells cocultured with control Mϕs. **(E,F)** Data represent means ± SEM of four independent experiments. Activation **(B)** and cytokine production **(C)** of the CD3 mAb-stimulated T cells was assessed by unpaired two-tailed *t*-test. Proliferation was statistically evaluated by two-way ANOVA with Bonferroni posttest **(E)** or one sample *t*-test **(F)**, respectively; *p < 0.05, **p < 0.01, ***p < 0.001.

Extracellular adenosine is well-known to dampen proinflammatory responses of immune cells, including Mϕs (35, 37, 44). To scrutinize the autocrine effects of adenosine generated from ATP by the Panx1/connexin-26/CD39/CD73-dependent mechanism in the immunoregulatory Mϕs, we treated Mϕs of both lineages with LPS + IFNγ and the CD39 inhibitor POM-1. After 2 days, we examined the expression of cytokine genes. Upon POM-1 treatment, we observed a more proinflammatory phenotype in the immunosuppressive M-CSF-differentiated subtype with enhanced *IL6, IL12B*, and *IL23A* expression. Further, *IL10* expression was reduced in three donors (Figure 6A). On the other hand, the highly expressed *IL6* and *IL23A* genes in the GM-CSF-differentiated subtype were rather downregulated by POM-1. Surprisingly, the highly expressed *TNF* was mildly downregulated in both Mϕ subtypes.

To further inspect the functional consequence of the CD39 blockade, we cocultured control and POM-1-pretreated Mϕs with autologous CD4^+^ T cells. POM-1 pretreatment of the M-CSF-differentiated/LPS + IFNγ-stimulated Mϕs abrogated their T cell-suppressive function, resulting in enhanced expression of the intermediate and late activation markers CD25 and CD147 on day 2 (Figure 6B), increased frequency of cytokine-producing T cells on day 5 (Figure 6C), and substantially enhanced T cell proliferation on day 7 (Figures 6D–F). In contrast, T cells cocultured with similarly treated GM-CSF-differentiated Mϕs produced lower amounts of IL-17A than T cells cocultured with Mϕs that were not treated with the CD39 inhibitor (Figure 6C). This correlated with POM-1-mediated changes of the *IL6* mRNA expression in the GM-CSF- and LPS + IFNγ-stimulated Mϕs (Figure 6A). Yet, the POM-1 pretreatment of this subtype had only minor effect on subsequent T cell proliferation, while pretreatment of all other Mϕ subtypes did not significantly affect T cells (Figures 6D–F). Thus, these data demonstrate that in response to the proinflammatory stimuli, M-CSF-differentiated Mϕs produce adenosine that skews them toward the immunoregulatory subtype, leading to T cell inhibition. In contrast, adenosine production by the GM-CSF-dependent Mϕs rather seems to potentiate their proinflammatory properties.

### FRβ^+^ Mϕs Co-Express CD39 and CD73 *In Vivo*

**Figure 7:**
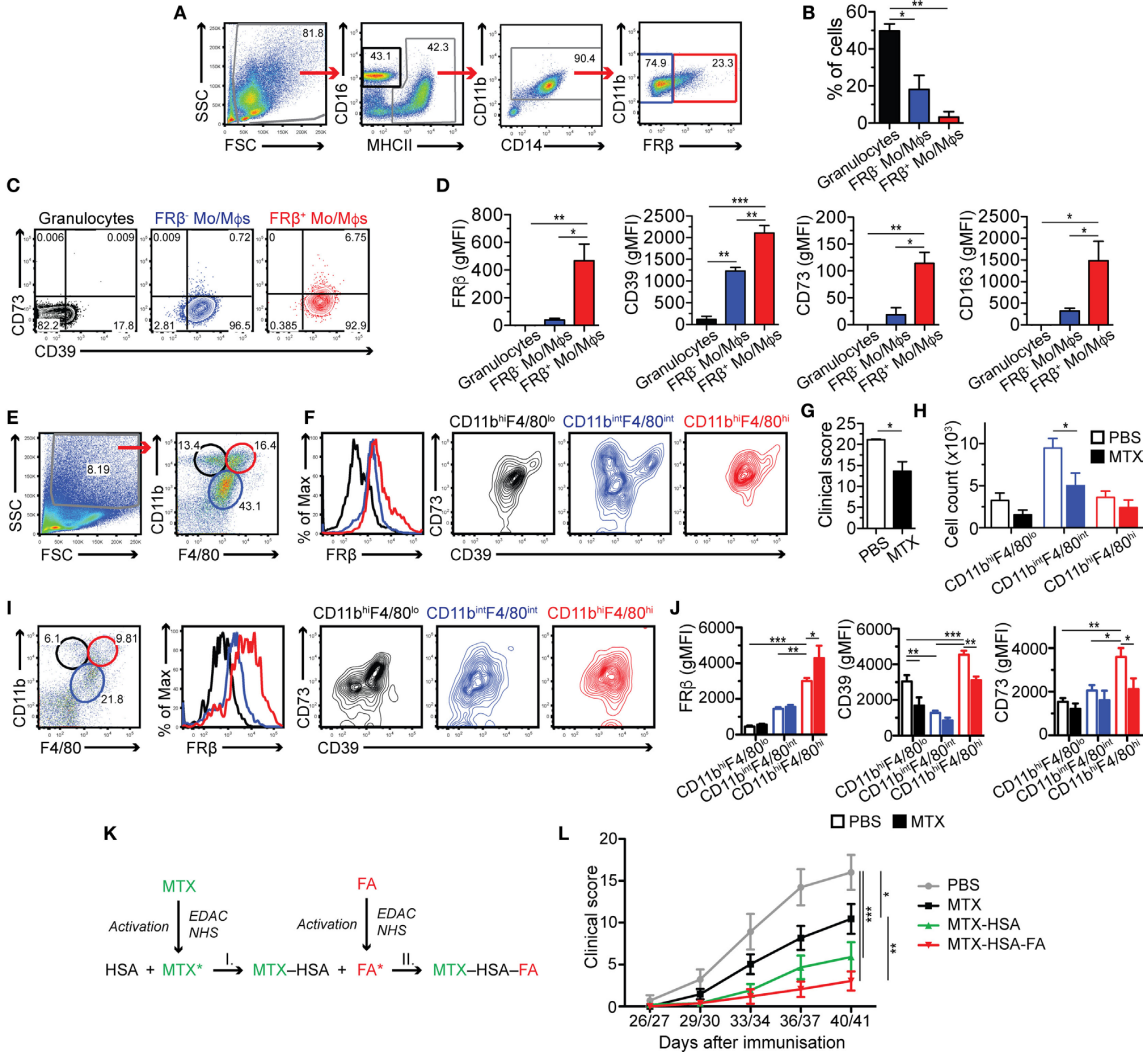
FRβ^hi^CD39^+^CD73^+^ macrophages (Mϕs) are present in arthritic joints of man and mouse and their targeting with methotrexate (MTX) suppresses joint inflammation. **(A)** Gating strategy to identify innate immune cell populations in the knee synovial fluid from arthritis-affected patients analyzed by flow cytometry. Granulocytes were identified as CD16^hi^MHCII^−/lo^ (black gate). MHCII^+^CD11b^+^CD14^+^ M-CSF-dependent monocytes/macrophages (Mo/Mϕs) were discriminated by prominent folate receptor β (FRβ) staining (red gate), while GM-CSF-dependent Mo/Mϕs were identified as FRβ^−^ (blue gate). **(B)** Frequency of CD16^hi^MHCII^−/lo^ granulocytes, FRβ^−^ and FRβ^+^ Mo/Mϕs in the synovial fluid of arthritis-affected patients. Data represent mean ± SEM of three patients. **(C)** CD39 and CD73 co-expression on granulocytes, FRβ^−^ and FRβ^+^ Mo/Mϕs present in the synovial fluid of arthritis-affected patients. One representative staining of three patients is shown. **(D)** FRβ, CD39, CD73, and CD163 surface expression [measured as the geometric mean of fluorescence intensity (gMFI)] in the three populations was statistically evaluated. Data represent mean ± SEM of three experiments. **(E)** Cells, isolated from ankles of arthritic DBA/1JRj mice, were analyzed by flow cytometry using the forward and side-scatter characteristics and CD11b and F4/80 expression. Three distinct populations were identified: CD11b^hi^F4/80^lo^ cells (black gate), CD11b^int^F4/80^int^ Mϕs (blue gate), and CD11b^hi^F4/80^hi^ Mϕs (red gate). **(F)** The populations were analyzed for FRβ (histogram on the left), CD39, and CD73 expression (contour plots on the right). **(G)** Clinical score of mice treated with PBS or MTX, respectively, on day 41. **(H)** Quantification of CD11b^hi^F4/80^lo^, CD11b^int^F4/80^int^, and CD11b^hi^F4/80^hi^ subsets isolated from ankles of PBS- or MTX-treated arthritic mice [representative dot plots shown in **(E,I)**] within the forward and side-scatter gate [shown in **(E)**, left]. **(I)** CD11b^hi^F4/80^lo^, CD11b^int^F4/80^int^, and CD11b^hi^F4/80^hi^ populations from MTX-treated arthritic mice (dot plot on the left), and their FRβ (histogram in the middle), CD39 and CD73 expression (contour plot on the right). One representative of two similar experiments of 3 + 3 mice; where each ankle was analyzed separately. **(J)** FRβ, CD39, and CD73 surface expression (measured as gMFI) shown in **(F,I)** was statistically evaluated. Data represent mean ± SEM of 3 + 3 mice with two ankles analyzed separately. **(K)** Conjugation strategy using *N*-(3-dimethylaminopropyl)-*N*-ethylcarbodiimide hydrochloride (EDAC) and *N*-hydroxysuccinimide (NHS), to create MTX-human serum albumin (HSA) conjugates (conjugation I) and MTX-HSA-folic acid (FA) conjugates (step I + II). **(L)** Mean clinical score (±SEM) of mice treated with various MTX formulations. Data are pooled from two independent experiments with 7–12 mice/group. The treatment and scoring in the second experiment was done one day earlier than in the first one. Statistical significance was assessed by one-way ANOVA **(B,D)**, unpaired two-tailed *t*-test **(G)** and two-way ANOVA **(H,J,L)**. *p < 0.05, **p < 0.01, ***p < 0.001.

To investigate whether adenosine-producing Mϕs play a role in inflammatory processes *in vivo*, we analyzed synovial fluid derived from patients with inflammatory arthritis by flow cytometry (Figure 7A). CD16^+^MHCII^−/lo^ granulocytes with weak CD39 expression represented the most abundant population in synovial fluid (Figures 7B,C). We used FRβ staining to discriminate between GM-CSF- and M-CSF-dependent monocytes/Mϕs (Figure 1A; Figure S1 in Supplementary Material) among the MHCII^+^CD11b^+^ cells. In line with our *in vitro* data, FRβ^+^ monocytes/Mϕs expressed significantly higher levels of CD39, CD73 as well as CD163 than FRβ^−^ monocytes/Mϕs (Figures 7C,D).

To further confirm our findings we analyzed myeloid cells from ankles of DBA/1JRj mice, in which we had induced arthritis using type II collagen (29). By co-staining with F4/80 and CD11b markers, we identified three distinct populations (Figure 7E): CD11b^int^F4/80^int^, CD11b^hi^F4/80^hi^, and CD11b^hi^F4/80^lo^ cells, the latter most likely corresponding to granulocytes. CD11b^int^F4/80^int^ Mϕs were most abundant and showed relatively low expression of FRβ and CD39 (Figure 7F). In contrast, CD11b^hi^F4/80^hi^ Mϕs, which are known to be M-CSF-dependent (45), were highly positive for FRβ, and, similarly to the human M-CSF-dependent Mϕs they co-expressed CD39 and CD73 (Figure 7F).

### Targeting of FRβ^+^CD39^+^CD73^+^ Mϕs With MTX Alleviates Arthritis

To ascertain whether adenosine produced by the FRβ^+^CD39^+^CD73^+^ Mϕ subset is implicated in control of joint inflammation, we treated the collagen II-immunized DBA/1JRj mice before arthritis onset (starting day 14 and then every 3–4 days) with the folate antagonist MTX. MTX exhibits an anti-rheumatic effect through inhibition of several enzymes involved in nucleotide synthesis, leading to release of adenine nucleotides to the extracellular space and their CD39 and CD73-dependent conversion to adenosine (46–48). Indeed, upon MTX treatment, we observed a reduction in the clinical score (Figure 7G) that was accompanied by the reduction of all monitored populations, with the CD11b^int^F4/80^int^ population affected the most by the MTX treatment (Figures 7H,I). In line with the *in vitro* data (Figures 4F,G), we detected that MTX treatment caused CD73 downregulation in all subsets, though only on the CD11b^hi^F4/80^hi^ subset the difference was significant. Additionally, we observed that CD39 was also expressed at lower levels (Figures 7I,J). These data demonstrate that MTX treatment is able to control inflammation in the arthritic joints and normalizes expression of adenosine-producing enzymes CD39 and CD73.

MTX can be transported into cells by FRβ, but its affinity to the receptor is ≈50 times lower in comparison to the prime FRβ ligand, FA (49). To specifically target FRβ^+^CD39^+^CD73^+^ Mϕs with MTX, we coupled both MTX and FA to HSA as carrier (MTX-HSA-FA; Figure 7K). As controls, we used free MTX or MTX conjugated to HSA (MTX-HSA), and compared the clinical benefit of the three MTX formulations in the CIA model. PBS-treated control mice developed severe arthritis over time (Figure 7L). As seen previously, MTX significantly reduced the clinical score of the arthritic mice compared to PBS treatment. MTX efficacy was further improved, but not significantly, by its coupling to HSA, which is best explained by a better retention of the HSA conjugates within inflamed tissues (50). Strikingly, the MTX-HSA-FA conjugate improved significantly the clinical score of the treated mice compared to free MTX. On the last evaluation day, the mean arthritic score of the MTX-HSA-FA-treated mice was reduced to one-third or half, compared to the MTX-treated or MTX-HSA-treated mice, respectively. Altogether, specific targeting of the adenosine-producing FRβ^+^CD39^+^CD73^+^ Mϕs with MTX potently alleviates the clinical signs of arthritis in the CIA mouse model.

## Discussion

**Figure 8:**
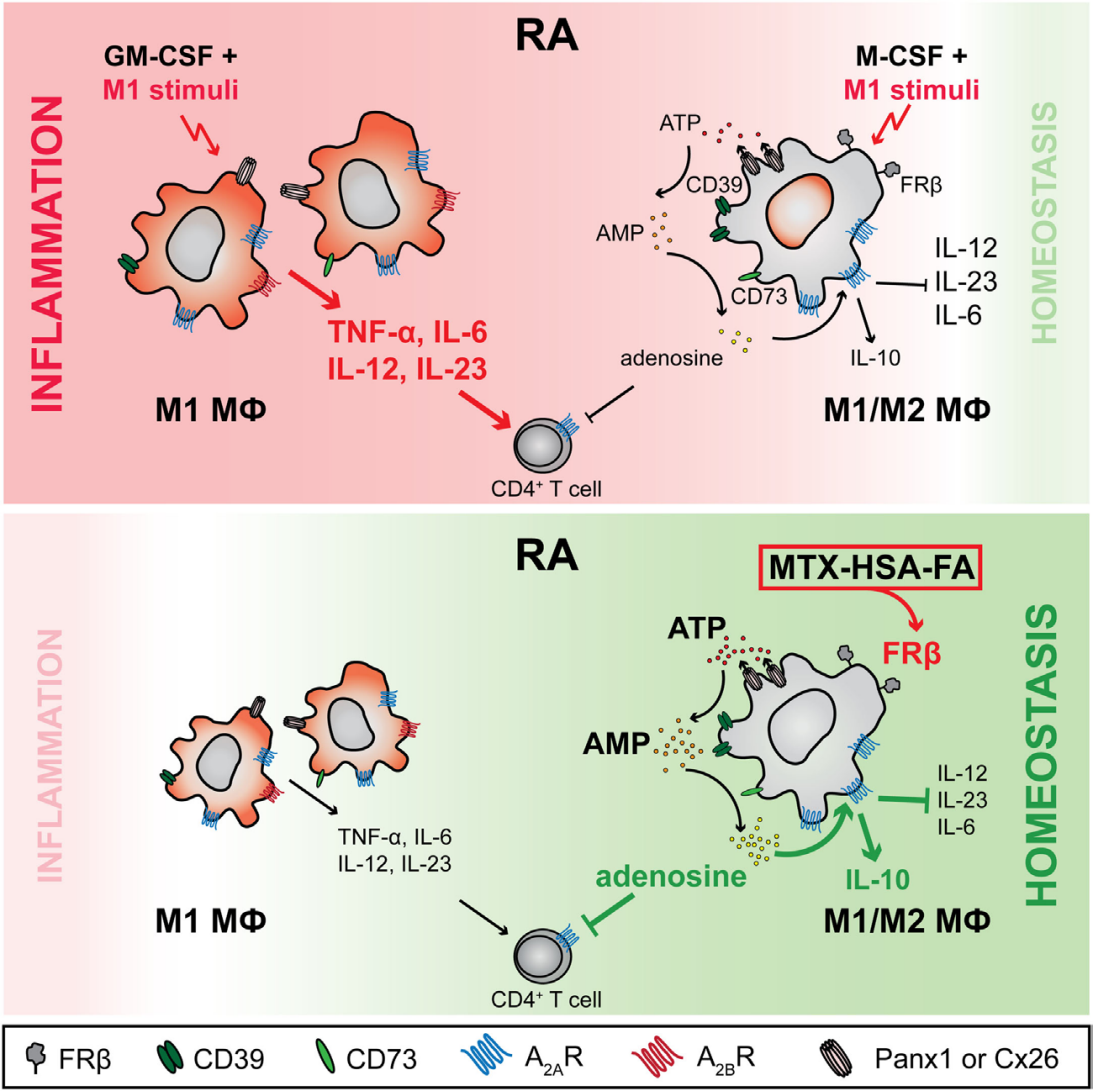
Working model—purinergic signaling differs in M-CSF- and GM-CSF-dependent macrophages (Mϕs): FRβ-targeted delivery of methotrexate (MTX) can restore homeostasis by boosting adenosine production in chronic inflammatory disorders. In rheumatoid arthritis (RA), Mϕs depending on Mϕ colony-stimulating factor (M-CSF) and expressing high levels of folate receptor β (FRβ) and CD39, in response to proinflammatory stimuli upregulate ATP-releasing channels pannexin-1 (Panx1) and connexin-26 (Cx26) as well as the ectonucleotidase CD73. As a direct consequence, this Mϕ subset produces high levels of extracellular adenosine, which acts in an autocrine fashion through adenosine 2A receptors (A_2A_Rs) to inhibit proinflammatory Mϕ responses. In particular, the release of proinflammatory cytokines (IL-6, IL-12, IL-23) is decreased while secretion of anti-inflammatory IL-10 is induced. Further, they suppress autoreactive T cells in a paracrine fashion. In contrast, adenosine production by the FRβ^−/lo^ Mϕs, the prevalent Mϕ species in RA joints, is inadequate due to a markedly lower expression of Panx1 and Cx26 and much reduced co-expression of CD39 and CD73. Furthermore, this GM-CSF-dependent Mϕ subset expresses the adenosine 2B receptor (A_2B_R), which triggers upon recognition of adenosine IL-6 and IL-23 production, leading to an enhanced Th17 response. As a result, only FRβ-targeted delivery of MTX to FRβ^+^CD39^+^CD73^+^ Mϕs *via* a MTX-HSA-FA conjugate boosts adenosine-mediated immune suppression and restores homeostasis.

Breaking self-tolerance leading to emergence of autoreactive T cells and autoantibodies is a hallmark of RA, suggesting that the disease is initiated by aberrant antigen presentation to T cells or aberrant antigen-specific T cell response (11, 51). Mϕs are the most abundant professional antigen-presenting cells in inflamed synovia (12), implying them as main drivers of pathogenic T cells. In the present study, we provide a comprehensive analysis of the interaction between Mϕs and T cells, which provides valuable insights in the molecular causes underlying perpetuating inflammation in RA-affected tissues. Based on our analysis, we also propose a therapeutic avenue to skew the balance toward resolution of inflammation (Figure 8).

To generate various Mϕ subtypes that resemble Mϕs present in RA tissues (12–16), and assess their effect onto T cell responses without contribution of bystander cells, we employed an *in vitro* coculture system of highly pure monocyte-derived Mϕs and autologous CD4^+^ T cells. We observed diametrically distinct Mϕ responses to proinflammatory stimuli (LPS + IFNγ), depending on the differentiation factor that was used for Mϕ generation. While GM-CSF-differentiated Mϕs were highly proinflammatory, M-CSF-dependent Mϕs, which were marked by pronounced and stable FRβ expression both in humans and mice, did not exhibit a classical M1 activation status, but rather a mixed M1/M2 phenotype seen also by others (52–54). Additionally, M-CSF-dependent LPS + IFNγ-stimulated Mϕs profoundly suppressed T cells, which is widely considered a prominent feature of M2 Mϕs (55–57). We demonstrated that this suppressive activity toward T cells as well as the M1-to-M2 shift was caused by the alteration of the Mϕ purinergic pathway leading to production and response to extracellular adenosine. The first product of the purinergic pathway, extracellular ATP released in response to TLR ligands, has been considered proinflammatory due to P2X7-dependent activation of the NLRP3 inflammasome and subsequent IL-1β and IL-18 release (58). However, Cohen et al. showed that extracellular ATP in mouse Mϕs acted in an anti-inflammatory manner due to the catabolic reaction provided by CD39 (40). We confirm and expand these data by showing that Mϕ CD39 is crucial for restraining early phases of T cell activation by local ATP degradation in the immunological synapse. However, CD39 degrades ATP to ADP and AMP only (37), and therefore, its catabolic activity must be paired with the AMP-degrading enzyme CD73, which we found to be upregulated by the proinflammatory stimuli and extracellular ATP, to efficiently produce adenosine. Mϕ-generated adenosine can then directly inhibit effector T cells by signaling through the high-affinity A_2A_ receptor that mediates most of the immunosuppressive effects of adenosine in immune cells by increasing intracellular cAMP (35, 37). Expression of the A_2A_ receptor in T cells is activation-induced (36, 42), which explains our observation that early T cell signaling was not entirely blocked, despite later inhibitory effects evidenced by lower expression of activation markers, cytokines and markedly reduced proliferation.

In addition to the effect on T cells, Mϕ-generated adenosine can also shape Mϕ phenotype via autocrine signaling and the effects of exogenously added adenosine or specific receptor agonists are well documented [reviewed in Ref. (35, 37, 44)]. Adenosine A_2A_ receptor stimulation inhibits TLR-mediated synthesis of TNF-α, IL-6 and IL-12, increases IL-10, VEGF, but also IL-1β (44, 59–63). On the other hand, signaling through the A_2B_ receptor alleviates TNF-α, IL-12 and potentiates IL-6 and IL-10 production (44, 64, 65). Alternatively, adenosine A_1_ and A_3_ receptor signaling leading to cAMP inhibition is thought to promote cell activation (35, 44). The cytokine profile of the immunosuppressive M-CSF-dependent Mϕ subtype is in line with adenosine signaling through the A_2A_ receptor, which we found highly expressed in response to the proinflammatory stimuli. Our experiments with the CD39 inhibitor POM-1 to block adenosine generation, resulting in increased *IL12B* and *IL6* (and decreased *IL10*) strengthened this hypothesis. The downregulation of the other adenosine receptors and nucleoside transporters by this Mϕ subset might account for the bias toward the immunoregulatory phenotype.

Although FRβ^−^ GM-CSF-differentiated Mϕs modulated expression of several genes of the purinergic pathway in response to LPS + IFNγ, adenosine production by this subset was low, either due to poor co-expression of CD39 and CD73, as reported for non-suppressive memory T cells (66, 67), or fast consumption through adenosine receptors and reuptake channels. POM-1-mediated downregulation of *IL6* expression further implied that GM-CSF-dependent Mϕs employed signaling *via* the A_2B_ receptor to enhance IL-6 levels upon stimulation with LPS + IFNγ, and skewed T cells in coculture experiments toward the Th17 lineage. In addition to *IL6*, POM-1 also inhibited *IL23A* expression in GM-CSF- and LPS + IFNγ-stimulated Mϕs, which together with IL-6 and either IL-1β or TGF-β, is necessary for the development of pathogenic Th17 cells (68, 69).

Interestingly, we found predominance of Mϕs with a phenotype similar to GM-CSF- and LPS + IFNγ-stimulated Mϕs (i.e., FRβ^−/lo^ and non-overlapping expression of CD39 and CD73) in arthritis-affected joints in both humans and mice. Based on our *in vitro* data, we presume that these Mϕs had developed in response to GM-CSF produced in high amounts by synovial CD4^+^ T cells (70). In humans, GM-CSF production is primarily linked to Th1 cells (70, 71). In mice, GM-CSF expression is directly confined to the Th17 subset (72, 73). Studies using autoimmune encephalomyelitis or myocarditis mouse models confirmed that GM-CSF produced by the Th17 subset is crucial for disease pathology by establishing a positive feedback loop *via* myeloid IL-23 and IL-6 that supports maintenance as well as *de novo* development of autoimmune Th17 cells (72–75). Therefore, it is not surprising that mice lacking *Csf2, Il23a, Il6* and *Il17* were shown to be protected from CIA (76–79), and blocking these genes or their receptors with mAbs is highly efficacious in RA patients enrolled in clinical trials (68, 80–82), in addition to the already approved IL6R mAb tocilizumab (82).

Based on our *in vitro* data, we propose that the balance between M-CSF- and GM-CSF-dependent Mϕ populations in tissues dictates whether the inflammation resolves through the action of adenosine or not. Indeed, in both humans and mice we observed that M-CSF-dependent FRβ^+^CD39^+^CD73^+^ Mϕs were present in arthritic joints at a low frequency and apparently were not sufficient to counteract the proinflammatory activity of the FRβ^−/lo^ GM-CSF-dependent Mϕs. Similarly, also the MTX treatment of our CIA mice was not able to completely suppress inflammation. It is conceivable that MTX is taken up by GM-CSF-dependent Mϕs *via* the highly expressed reduced folate carrier/Slc19a1 (83, 84), and then promotes as a consequence inflammation through upregulation of *IL6* and *IL23A*. Therefore, only folate-directed delivery of MTX through the HSA-MTX-FA conjugate into adenosine-producing FRβ^+^CD39^+^CD73^+^ Mϕs was able to control the inflammation in the CIA model. These data are further supported by our recent results showing that mice treated with folate-functionalized liposomes with MTX shielded in the liposome cavity were completely protected from CIA (29).

In conclusion, we have demonstrated that extracellular purine metabolism governs the switch from the proinflammatory to the suppressive Mϕ phenotype. This newly discovered mechanism provides a rationale for specific targeting of the purine metabolism by modulating drugs, such as MTX, in order to fully resolve Mϕ-driven diseases including inflammation of RA.

## Ethics Statement

The study using human material was performed in accordance with the Declaration of Helsinki, informed consent was obtained from all participants and research was approved by the Ethics Committee of the Medical University of Vienna (2177/2013, 559/2005). The experiments using mouse models were approved by the French Ministry of Research and the Paris Descartes University Ethical Committee (CEEA No. 34); agreement No. CEEA34.GB.029.11. All methods and experiments were performed in accordance with the relevant guidelines and regulations.

## Author Contributions

AO-R and HS designed, performed or supervised research, and wrote the manuscript, CM, RP, VL, GZ, and JH performed or supervised research and provided feedback, GM measured adenosine, CC, DR, FL, VF, GR, and GB designed and performed mouse experiments, including HSA-MTX-FA conjugation, and provided feedback, TB analyzed microarray data, SB, TM, MB, MS, MF, and AC-P provided materials and feedback. All authors approved the manuscript.

## Conflict of Interest Statement

MB and MS are employed by EXBIO Praha. All other authors declare that the research was conducted in the absence of any commercial or financial relationships that could be construed as a potential conflict of interest.

## Supplementary Material

Movie S1 Related to Figure 5C. Live cell imaging of the human CD4^+^ T cell interaction with an autologous CD39^+^CD73^−^ M-CSF-differentiated and LPS + IFNγ-activated macrophage (Mϕ), which showed enriched expression of CD39 in the immunological synapse over time. Time-lapse microscopy of Mϕ-expressed CD39 (shown in green), its recruitment to the synapse and T cell calcium flux monitored by Fluo-4 fluorescence (in pseudocolor; range is depicted in Figure 5C) over time. Images were acquired every minute for 45 min.

Movie S2 Related to Figure 5D. Live cell imaging of the human CD4^+^ T cell interaction with an autologous CD39^+^CD73^−^ M-CSF-differentiated and LPS + IFNγ-activated macrophage (Mϕ), which did not show enriched expression of CD39 in the immunological synapse over time. Images were acquired every minute for 43 min. CD39 staining on the M-CSF-differentiated and LPS + IFNγ-activated Mϕ is shown in green, while fluorescent calcium sensor Fluo-4 in T cells is shown in pseudocolor.
