# Supplementary material for: Extracellular Purine Metabolism Is the Switchboard of Immunosuppressive Macrophages and a Novel Target to Treat Diseases With Macrophage Imbalances
Source: Front Immunol. 2018 Apr 27;9:852. doi: 10.3389/fimmu.2018.00852 (PMC5946032; doi:10.3389/fimmu.2018.00852)
Supplement: Supplementary file 3 [file presentation_1.PDF]

Supplementary Material

Extracellular Purine Metabolism Is the Switchboard of Immunosuppressive Macrophages and a Novel Target to Treat Diseases with Macrophage Imbalances

Anna Ohradanova-Repic*, Christian Machacek, Celine Charvet, Franck Lager, Delphine Le Roux, René Platzer, Vladimir Leksa, Goran Mitulovic, Thomas R. Burkard, Gerhard J. Zlabinger, Michael B. Fischer, Vincent Feuillet, Gilles Renault, Stephan Blüml, Miroslav Benko, Miloslav Suchanek, Johannes B. Huppa, Takami Matsuyama, Artur Cavaco-Paulo, Georges Bismuth, Hannes Stockinger*

*** Correspondence:** Anna Ohradanova-Repic: anna.repic@meduniwien.ac.at, Hannes Stockinger: hannes.stockinger@meduniwien.ac.at

1 Supplementary Figures and Tables

Table S1. Primers used in this study

Gene symbol	Forward primer (5'➔3')	Reverse primer (5'➔3')
ACTB	ATTCCTATGTGGGCGACGA	GCGTACAGGGATAGCACAGC
EEF1A1	GTGCTAACATGCCTTGGTTC	AGAACACCAGTCTCCACTCG
ADORA1	CCTCATCCTCACCCAGAGC	TCAGTCCCACCACGAAGG
ADORA2A	TCTTCATTGCCTGCTTCGTC	GATGCCCTTAGCCCTCGT
ADORA2B	CTCCATCTTCAGCCTTCTGG	AGGACAGCAATGACCCCTCT
ADORA3	GGACTTTGCCAGGGATGAC	CCTTGCAGCTTCTGGTTTTG
PANX1	GCTGCCTTTGTGGATTCATATT	CAGCAGGATGTAGGGGAAAA
GJB2	TCCCGACGCAGAGCAAA	CAGCCACAACGAGGATCATAA
SLC28A3	AAAGAGCATGGAGCTGAGGAG	CCTGTTTGGTGTTTGTGTGC
SLC29A3	AAACTCCGCAACTCCTCCA	CACGGATGTGGACTGCAAC
ADA	CCTTCGACAAGCCCAAAGTAG	CAGCCCCTCTGCTGTGTTAG
IL10	TCCCTGTGAAAACAAGAGCAAG	AGTCGCCACCCTGATGTCTC
IL12B	CATCTCTTGGTTTTCCCTGGT	AGCCTAAGACCTCACTGCTCTG
IL23A	GCTGGGGAGCAGAGCTGTA	TCTCTTAGATCCATGTGTCCCACT
IL6	TCACTGGTCTTTTGGAGTTTGAG	GTTGGGTCAGGGGTGGTTAT
TNF	ACGCTCTTCTGCCTGCTG	CTTGTCACTCGGGGTTCG

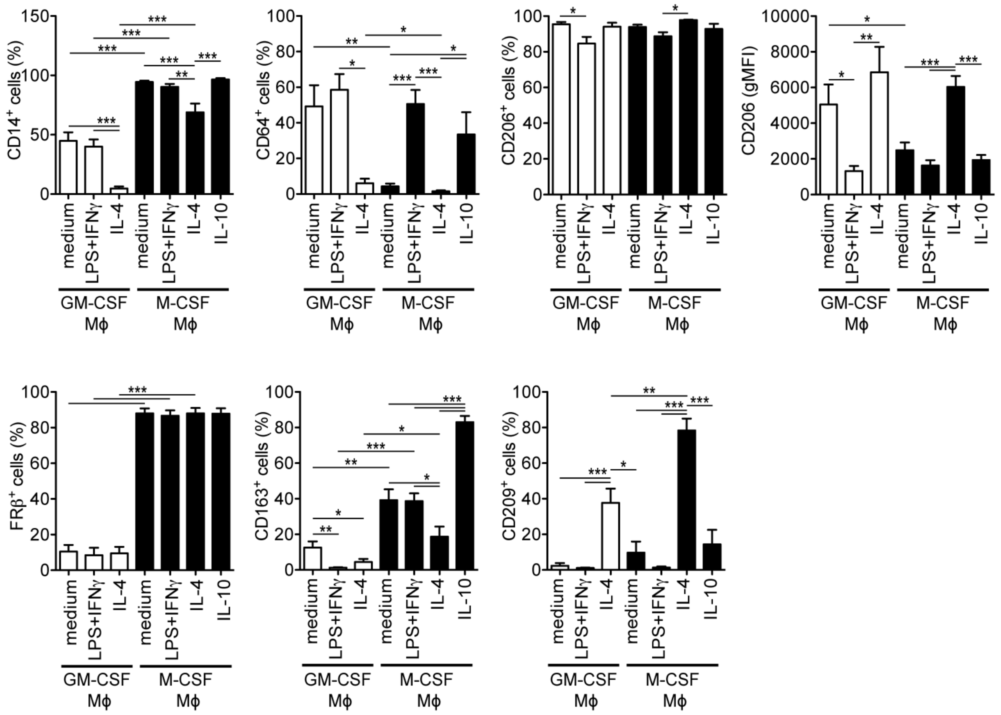

Figure S1. Phenotype of activated human GM-CSF- and M-CSF-differentiated macrophage subtypes. Statistics of surface expression of macrophage markers, shown in Figure 1A. The mean percentages of marker-positive cells ± SEM from 10-12 donors are shown. For highly expressed CD206 also the geometric mean of fluorescence intensity (gMFI) ± SEM is shown. *$p<0.05$, **$p<0.01$, ***$p<0.001$. Statistical significance was determined by one-way ANOVA with Tukey post-test.

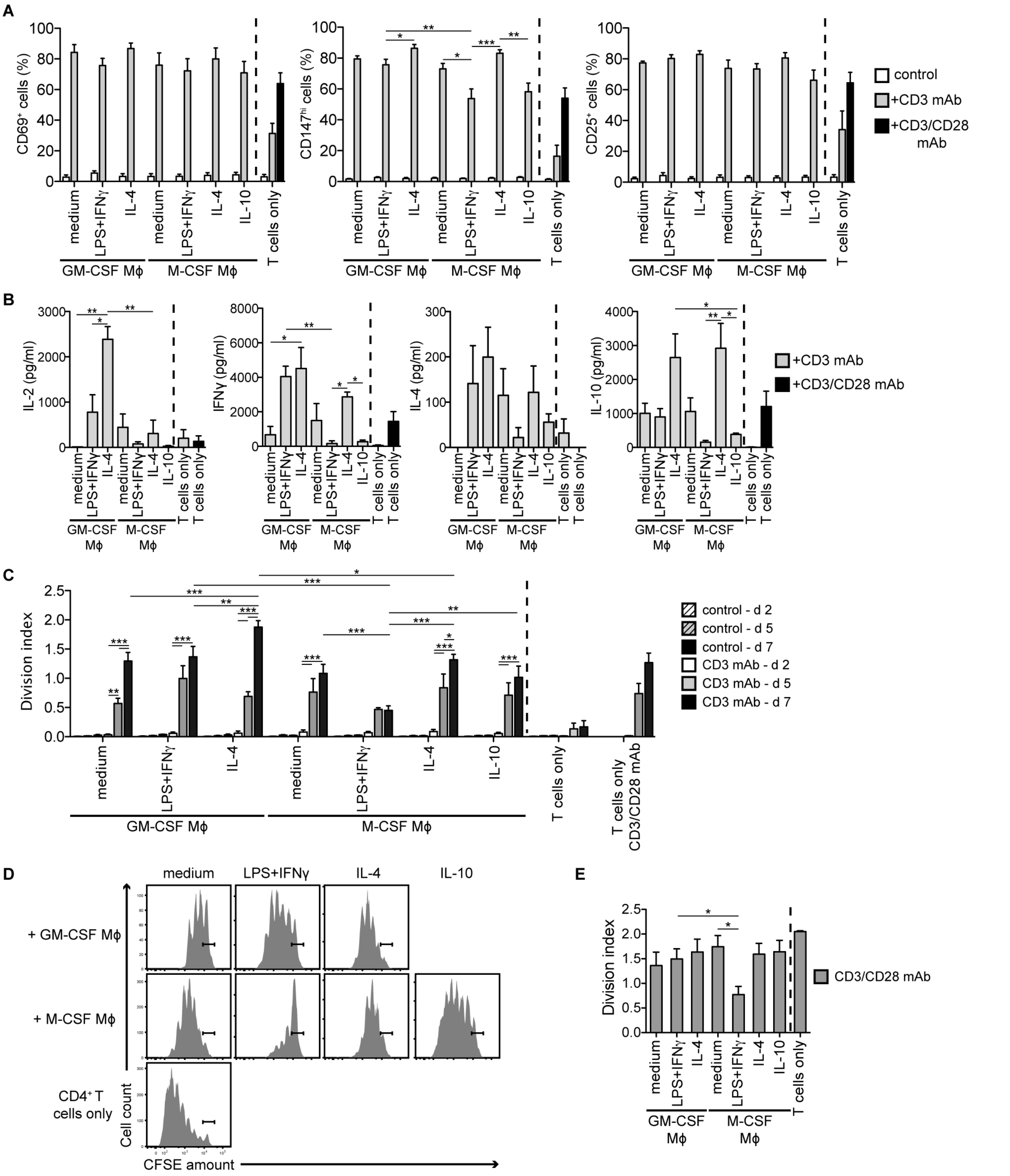

Figure S2. M-CSF-differentiated macrophages activated with LPS+IFNγ suppress activation, cytokine production and proliferation of stimulated autologous CD4$^+$ T cells. Macrophages were differentiated for 7 d with either GM-CSF or M-CSF, then activated for 2 d with the indicated stimuli and afterwards cocultured with autologous CD4$^+$ T cells in the presence or absence of stimulating mAbs as indicated (CD3 mAb or CD3+CD28 mAb). As a control, T cells were cultured alone as

indicated. (**A**) To measure the extent of T cell activation, CFSE-labeled $CD4^+$ T cells were stained for the activation markers CD69 and CD147 after 2 d of coculture (shown in Figure 2A) and the percentages of positive cells (or highly positive cells, in case of CD147) from 8 independent experiments were expressed as mean ± SEM. Additionally, CD25 was used in four experiments. (**B**) Cell culture supernatants from 2-day macrophage-T cell co-cultures in the presence of CD3 mAb OKT3, and T cells alone activated with CD3±CD28 mAb were analyzed for IL-2, IFNγ, IL-4 and IL-10. The mean cytokine concentrations ± SEM from 3 donors are shown. (**C**) Proliferation of CFSE-labeled T cells from 4 donors co-cultured with autologous macrophages ± CD3 mAb was determined by CFSE dilution on day 2, 5 and 7 using flow cytometry. (**D, E**) Cell proliferation analysis of CFSE-labeled autologous $CD4^+$ T cells after 7 d coculture with the different macrophage subsets in the presence of CD3+CD28 mAbs. One representative experiment, where $CFSE^{hi}$ non-dividing cells are gated (D), and statistics from of 4 independent experiments (E) is given. Expression of activation markers (A), cytokine production (B) in the CD3 mAb-stimulated T cells in cocultures were statistically evaluated by one-way ANOVA with Tukey post-test. Proliferation was assessed by two-way ANOVA with Bonferroni post-test (C) or one-way ANOVA with Tukey post-test (E); *$p<0.05$, **$p<0.01$, ***$p<0.001$.

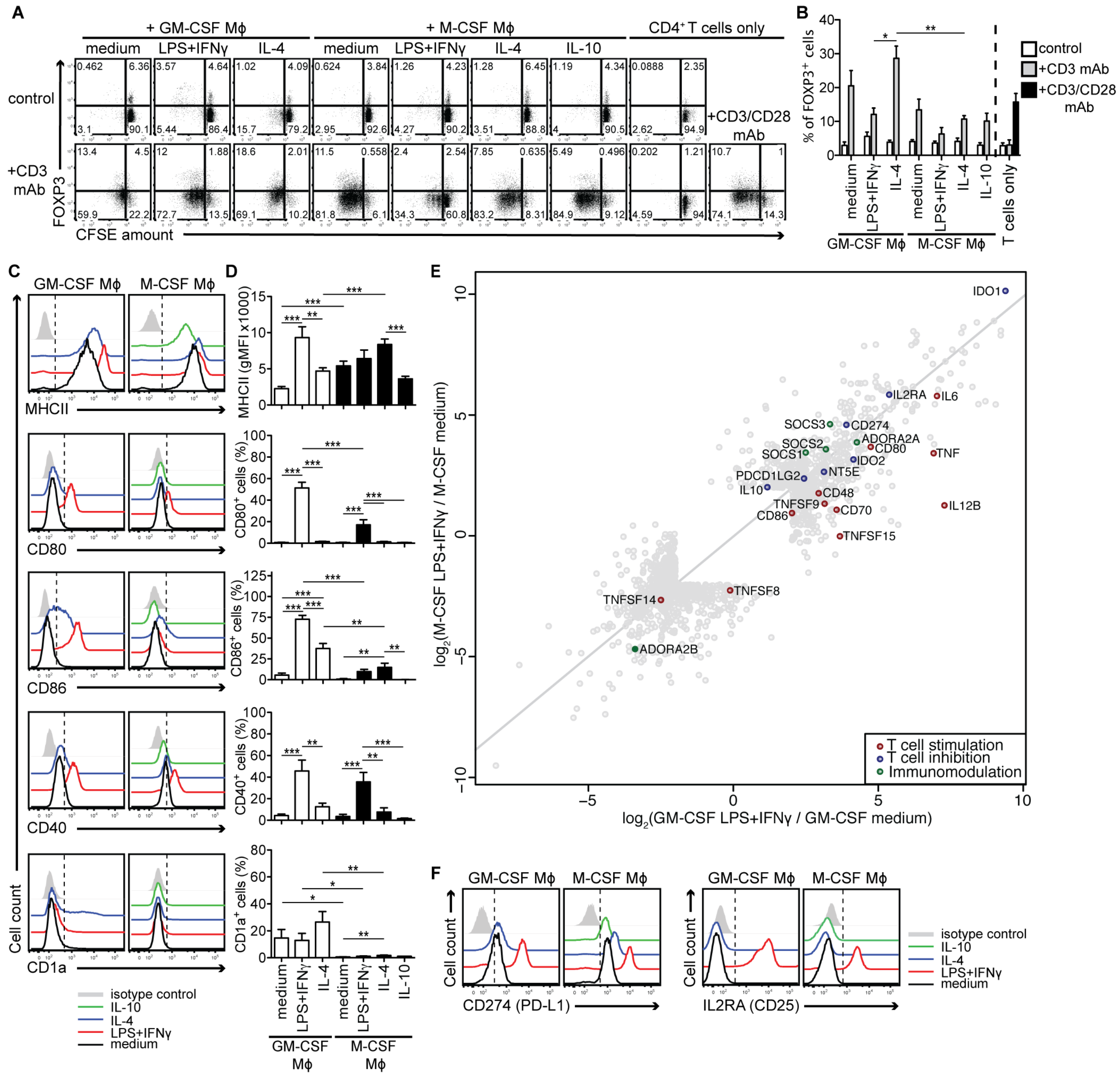

Figure S3. Dissecting molecular mechanisms underlying the suppressor macrophage phenotype. (**A, B**) Macrophage-T cell co-cultures were set up as described in Figure 2. On day 6, CFSE-labeled CD4^{+} T cells were stained for FOXP3 to measure the percentage of Tregs. One representative experiment (A) and mean percentages of FOXP3^{+} T cells from 5 independent experiments (B) are shown. (**C, D**) Surface expression of MHC class II, CD1a and costimulatory molecules CD80, CD86, CD40 on macrophage subsets described in Figure 1 was analyzed by flow cytometry. One representative experiment (C) and the mean percentages of positive cells ± SEM from 10-12 donors (D) are shown. Since all macrophage types were >95% positive for MHC II, geometric mean of fluorescence intensity (gMFI) is given instead. (**E**) Scatterplot of the difference in the mean expression of 1503 genes that were significantly changed at least 4-fold ($\log_2$ difference >2; with adj. p value <0.05) upon LPS+IFNγ stimulation in either GM-CSF-differentiated or M-CSF-differentiated macrophages. Multiple probe sets per gene that passed the selection criteria (total

2633) were averaged. The diagonal line indicates equal regulation by LPS+IFNγ in both macrophage lineages. Data are from gene expression profiling of macrophages from 3 donors differentiated and activated as described in Figure 1. (**F**) Surface expression of PD-L1 and CD25 in the different macrophage subsets described in Figure 1 was analyzed by flow cytometry (n=2 for PD-L1, n=4 for CD25). CD3 mAb-driven T cell FOXP3 expression from cocultures (B) and macrophage marker expression (D) was evaluated by one-way ANOVA with Tukey's posttest; *$p<0.05$, **$p<0.01$, ***$p<0.001$.

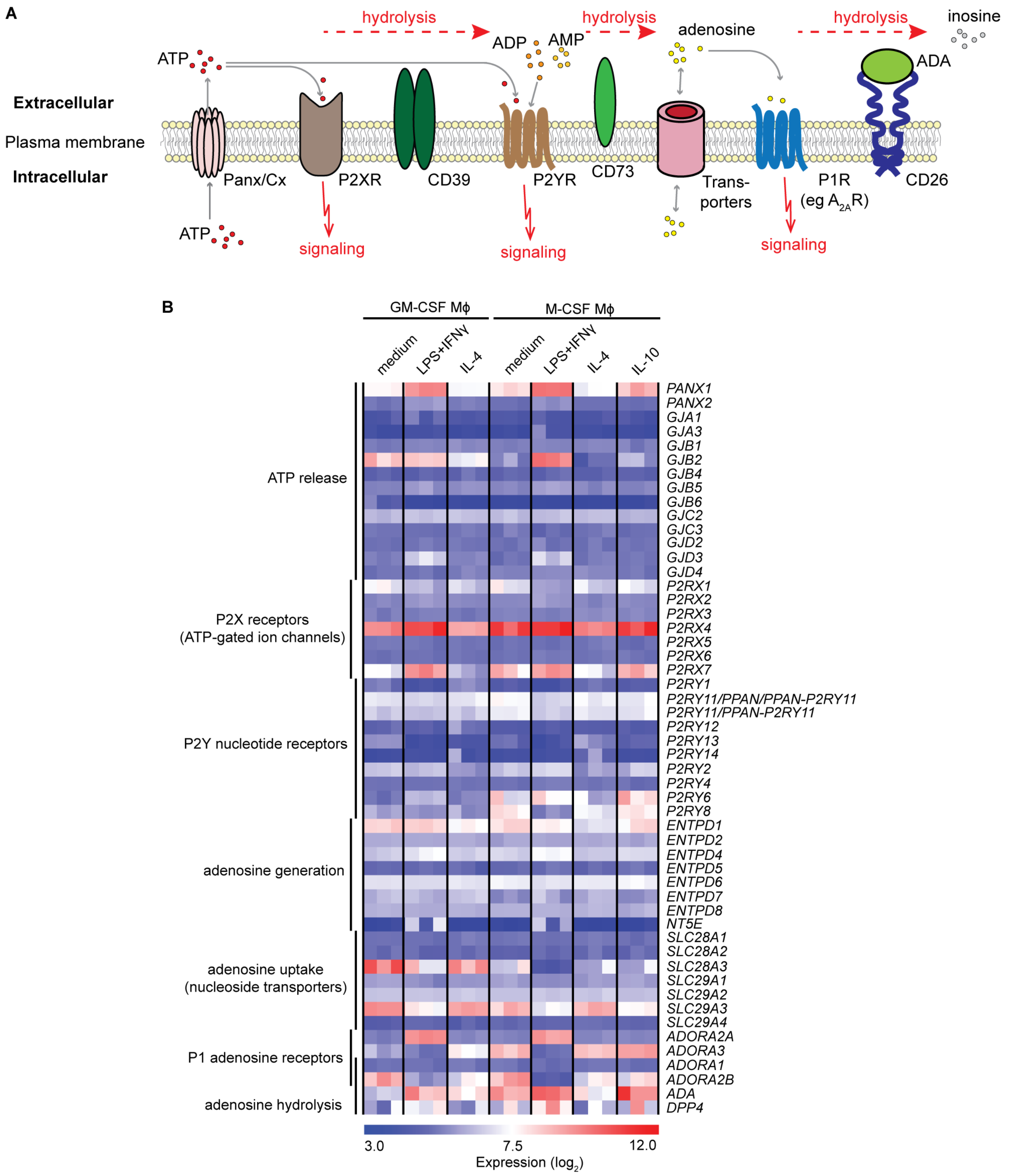

Figure S4. Schematic overview of the purinergic pathway and expression of genes mediating purinergic signaling in human macrophage subtypes. (**A**) Components of the purinergic pathway. Upon stimulus, pannexin (Panx) or connexin (Cx, encoded by the *GJA-D* genes) channels open and release ATP from the cell. Extracellular ATP promotes activation of P2 receptors (encoded by the *P2RX* and *P2RY* genes). Ectonucleotidases, encoded by the *ENTPD* genes, such as CD39 (*ENTPD1*) work in tandem with the ecto-5´-nucleotidase CD73 (encoded by the *NT5E* gene) to hydrolyze ATP

to adenosine, which activates P1 receptors (encoded by the *ADORA* genes). Adenosine is degraded by adenosine deaminase to inosine or recycled through concentrative nucleoside transporters (CNTs, encoded by the SLC28A genes) or equilibrative nucleoside transporters (ENTs, encoded by the SLC29A genes). Usually intracellularly localized enzyme adenosine deaminase (ADA) can be attached to the cell surface via transmembrane protein CD26 (encoded by *DPP4*) or alternatively, via A_1R and $A_{2B}R$ adenosine receptors. (**B**) Macrophages of 3 donors were differentiated and activated as detailed in Figure 1. Afterwards, their gene expression was determined by microarray profiling. Heat map of transcripts for genes involved in ATP release, metabolism and purinergic signaling or uptake based on all gene probe sets with $\log_2$ expression >5 in at least one sample; for genes with duplicate probe sets, their mean values were plotted. Each donor is visualized separately.

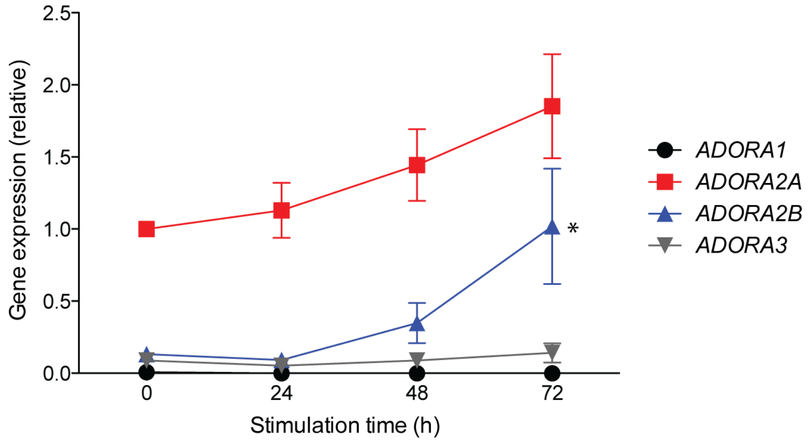

Figure S5. Expression of adenosine receptor genes in human T cells. Human $CD4^+$ T cells were stimulated with plate-bound CD3 mAb OKT3 (1 µg/ml) plus soluble CD28 mAb L293 (0.5 µg/ml) for 24-72 h and RNA was harvested. As a control, RNA was harvested prior to stimulation. mRNA levels of the adenosine receptors and of the housekeeping gene *EEF1A1*, which was a stable endogenous control in blasting T cells, were measured by qRT-PCR. The data were normalized to the levels of the *ADORA2A* gene in non-stimulated T cells that were set to one. Data represent mean ± SEM of 4 independent experiments. Expression of each receptor gene was evaluated by one-way ANOVA with Tukey post-test; *$p<0.05$.
